# Synthesis, Characterization, and BSA Binding Properties of Carboxylated Merocyanine-Based Fluorophores

**DOI:** 10.1021/acsomega.4c07997

**Published:** 2024-11-26

**Authors:** Rodrigo C. Duarte, Rodrigo Cercená, Bruno B. de Araujo, Otávio A. Chaves, Paulo F. B. Gonçalves, Eduardo Zapp, Fabiano S. Santos, Fabiano S. Rodembusch, Alexandre G. Dal-Bó

**Affiliations:** † Universidade do Extremo Sul Catarinense (UNESC), Av. Universitária, 1105, Criciúma CEP 88806-000, SC, Brazil; ‡ Instituto de Química, Universidade Federal do Rio Grande do Sul (UFRGS), Porto Alegre CEP 91501-970, Brazil; § CQC-IMS, Department of Chemistry, University of Coimbra, Rua Larga s/n, 3004-535 Coimbra, Portugal; ∥ Departamento de Ciências Exatas e Educação (CEE), Universidade Federal de Santa Catarina, Blumenau 89036-004, Brazil

## Abstract

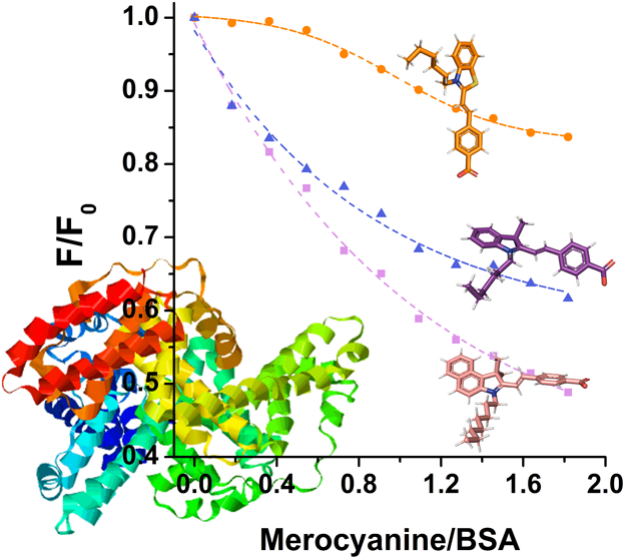

This study describes the synthesis of new carboxylated merocyanine dyes by Knoevenagel condensation between 4-carboxybenzaldehyde and indolium/benzoindolium- and benzothiazolium-based coupling compounds. The condensations were performed in the presence of ammonium acetate, and the products were obtained in good yields after simple purification. These merocyanines exhibit UV-A-to-blue absorption and blue-to-green fluorescence emission, characterized by relatively large Stokes shift values (∼5000 cm^–1^). In addition, quantum chemical calculations were conducted to better explore the electronic and photophysical properties of the merocyanines under study. Thermal analysis via thermogravimetric analysis (TGA) revealed distinct decomposition stages for the merocyanines, with stability up to 200 °C. Cyclic voltammetry revealed irreversible waves for donor oxidation and acceptor reduction. On the basis of the onset potentials, the highest occupied molecular orbital (HOMO) energies were estimated to be between −5.38 and −5.47 eV, and the lowest unoccupied molecular orbital (LUMO) energies were calculated to range from −3.20 to −3.24 eV. These values suggest a narrow electrochemical band gap of 2.07 to 2.13 eV. Finally, fluorescence quenching experiments using the intrinsic fluorescence of the Trp residues in BSA were successfully applied to these compounds, indicating strong interactions with this protein via a static mechanism. The docking simulations corroborated the interaction between the merocyanines and BSA.

## Introduction

Merocyanine dyes are an important class of cyanine compounds with a nonsymmetrical architecture and electron donor–acceptor side groups. The different electronic features of these side groups play a significant role in their electronic structure, equilibrium structure, and electronic spectra.^1,2^ These groups are represented by an electron donor nitrogenous heterocycle and an electron acceptor group, normally oxygenated, separated by a polymethine chain with an even number of carbon atoms.^3,4^ These compounds can assume neutral or charged configurations and are strongly influenced by physical-chemical and structural factors. These structures offer ample possibilities for the modification of photophysical systems and photochemical properties and strongly depend on the polarity of the medium.^5^ Based on these features, they can be classified into 3 different groups according to the size of the polymethine chain: (i) simple merocyanines, (ii) merocarbocyanines, and (iii) meropolycarbocyanines. These structures coexist in zwitterionic charged species.^6,7^ In addition, changes in the color of these compounds in response to the environment and the acid–base character of merocyanine are highly important for obtaining new pH and anion sensors.^8,9^ Changes in polarity, viscosity, and the association or formation of complexes with certain analytes significantly affect the photophysical properties of these compounds since the observed changes can be used as analytical responses and can therefore be used as optical sensors,^10−12^ although different uses have also been reported, including the control of supramolecular assemblies,^13^ molecular switches,^14^ microbial fuel cells,^15^ and nanoparticle assemblies.^16^

On the other hand, comprehending drug binding to plasma proteins, particularly human serum albumin (HSA), is vital for pharmacodynamics and pharmacokinetics.^17,18^ HSA plays a pivotal role in transporting and storing both endogenous and exogenous compounds and maintaining blood homeostasis.^19,20^ For investigation purposes, bovine serum albumin (BSA) is often favored as a substitute for human serum albumin (HSA) owing to its cost-effectiveness and widespread availability. Notably, BSA shares substantial protein sequence similarity with HSA, exhibiting 76% identity and 88% similarity, suggesting that the two proteins have comparable binding capacities.^21^ This implication has led to the extensive utilization of spectroscopic techniques for investigating the interplay between synthetic compounds and serum albumins. In terms of electronic spectroscopy, both HSA and BSA proteins exhibit robust fluorescence emission upon excitation at approximately 280 nm, with the peak intensity observed at approximately 334 nm. This fluorescence primarily arises from tryptophan residues, which are usually responsible for their intrinsic fluorescence. The interaction of small molecules with BSA typically quenches the fluorescence emission from serum albumin, a phenomenon well-established through various studies.^22,23^ However, despite the numerous reported applications of merocyanines, studies on their interactions with albumins are limited,^24−26^ except for merocyanine MC 540 (MC540).^27−30^ Merocyanine 540 (MC 540) is a member of the benzoxazol merocyanine dye family and is characterized by heterocyclic aromatic groups connected via a polymethine chain. The optical properties of these dyes are highly responsive to variations in environmental conditions, including polarity, viscosity, and temperature.^31^ In general, MC540 interacts strongly with bovine serum albumin (BSA) and human serum albumin (HSA), forming ground state complexes and increasing its fluorescence.^32^ The binding is entropy-driven and occurs at multiple sites, with HSA showing greater structural flexibility.^32,33^ MC540 adsorption on HSA leads to dye monomerization, increasing fluorescence and triplet lifetimes while reducing photoisomerization rates. Compared with BSA, HSA is more strongly associated with MC540.^34^

This study reports the synthesis of new carboxylated merocyanines through Knoevenagel condensation. The selected compounds present different nitrogen-quaternized heterocycles, as well as different alkyl chains, offering new insights into the structure–property relationships of this class of compounds. Considering the crucial role of merocyanine dyes in sensing and photophysical applications, we also provide a comprehensive analysis of their photophysical, thermal, and electrochemical properties. In addition, on the basis of the well-established interaction between certain merocyanines such as MC540 and serum albumins, exploring the interaction of a new cyanine dye with bovine serum albumin (BSA) could provide valuable information into the potential of the dye as an optical sensor and its binding characteristics, thereby contributing to the development of novel sensor technologies and expanding our understanding of protein–dye interactions.

## Results and Discussion

### Synthesis and Characterization

**Scheme 1:**
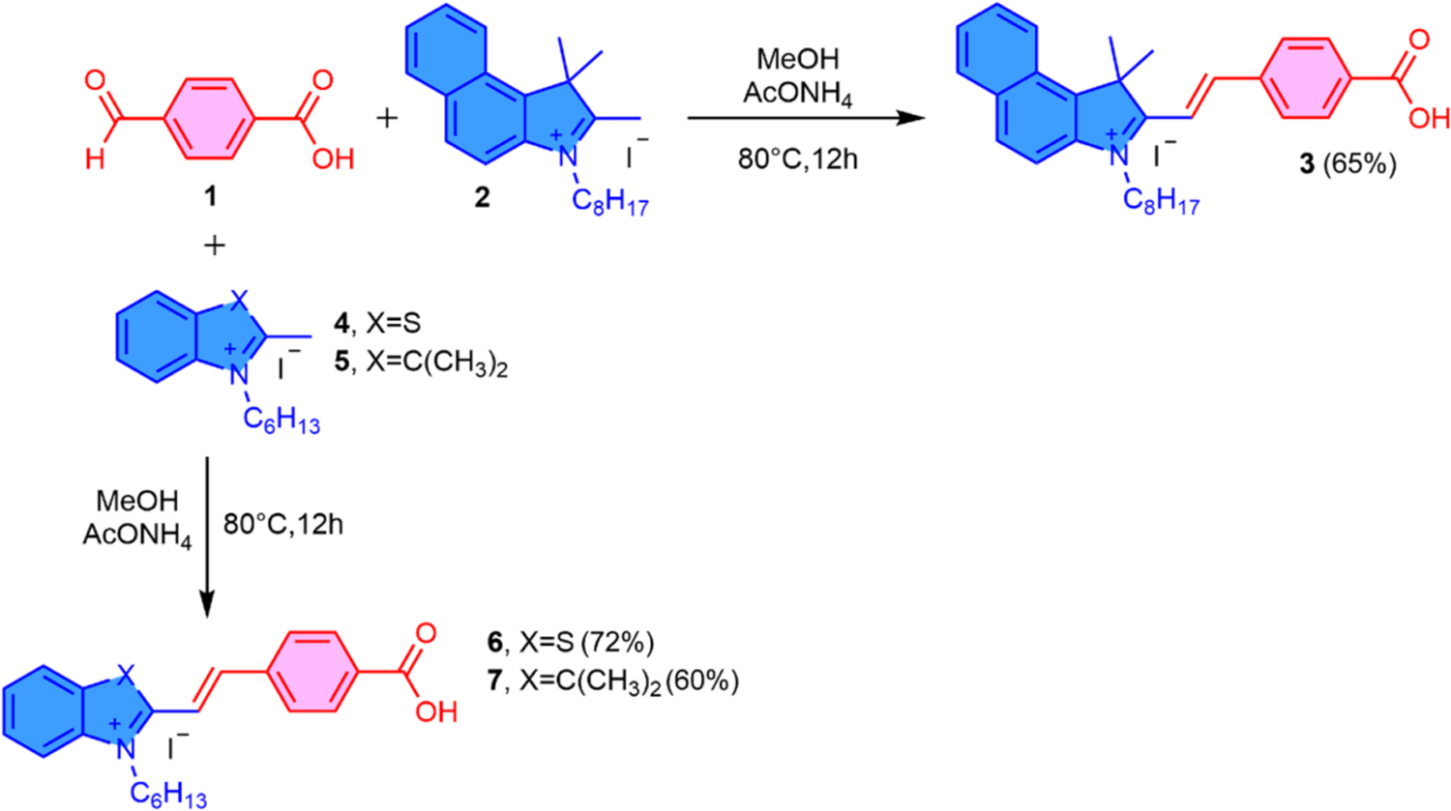
Synthesis of Merocyanines **3**, **6**, and **7**

The nitrogen-quaternized compounds **2**, **4**, and **5** (benzoindolium, benzothiazolium, and indolium derivatives, respectively) were synthesized following established procedures reported in the literature.^35,36^ Merocyanines **3**, **6**, and **7** were subsequently obtained through a Knoevenagel condensation. This reaction involved the coupling of 4-formylbenzoic acid (**1**) with quaternized heterocycles **2**, **4**, and **5** in a basic medium. Methanol was used as the solvent to ensure the complete solubility of the reactants, and the reaction mixture was maintained under reflux for 12 h to achieve optimal product formation (Scheme 1).^37^ After the solvent was evaporated, the resulting solid was washed with dichloromethane and then dried under vacuum to yield the respective products as colored solids in moderate yields. Notably, the ^1^H NMR spectra (see the SI) of the merocyanines exhibit two doublets, which are assigned to olefinic protons. The observed coupling constant (*J*) of 16.0 Hz supports the presence of the trans conformer, as proposed.

**Figure 1:**
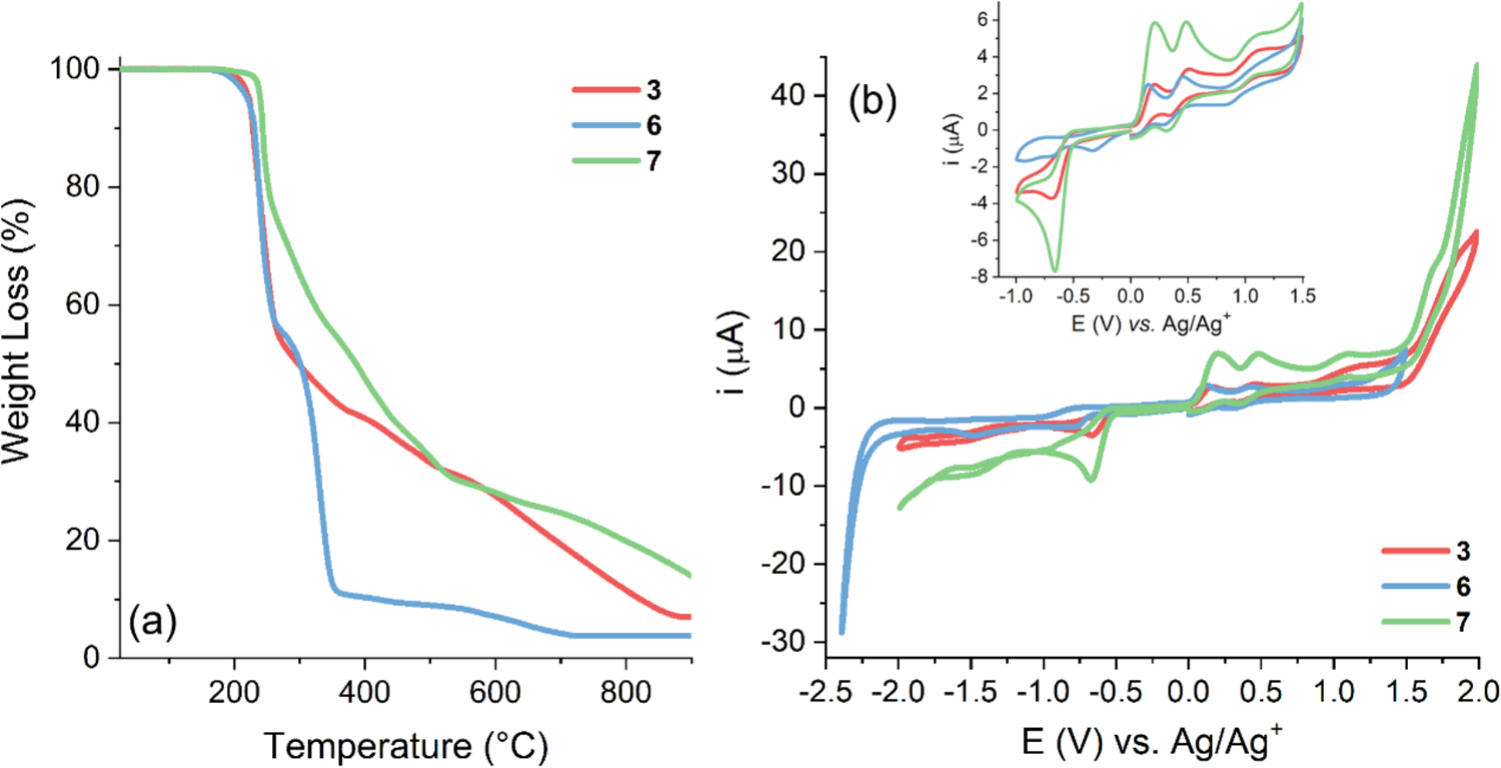
(a) TGA curves of merocyanines **3**, **6**, and **7** under a N_2_ atmosphere at a heating rate of 10 °C·min^–1^. (b) Cyclic voltammograms of the same compounds obtained in 0.01 M TBAPF_6_ in CH_2_Cl_2_ at a scan rate of 100 mV·s^–1^. Inset: Processes obtained in the potential range between −1.0 and 1.5 V vs Ag/Ag^+^.

The thermal, electrochemical, and photophysical properties of the studied compounds were evaluated. To expand the data on the thermal stability of this class of compounds and assess their stability for potential applications involving large temperature variations, the thermal properties of merocyanines **3**, **6**, and **7** were determined by thermogravimetric analysis (TGA) under a N_2_ atmosphere. Figure 1 shows that the thermogravimetric curves could be divided into at least three stages of decomposition. Merocyanines **3** and **7** showed overlapping weight loss curves in the range of 270–800 °C, hindering the separation of step degradation and the identification of the molecular components involved in degradation. The samples were thermally stable, with initial decomposition temperatures in the range of 219–236 °C (**3**: 219 °C, **6**: 226 °C, and **7**: 236 °C). In general, the first thermal event occurred at 300 °C, with a mass loss of approximately 50% for merocyanines **3** and **6**. In contrast, for merocyanine **7**, a weight loss of approximately 20% occurred, followed by an additional weight loss of ∼30% at temperatures up to 400 °C. This weight loss could be related to the degradation of the quaternized heterocycle in these three cases. The other thermal events are quite different depending on the merocyanine, with a mass loss of approximately 40% observed between approximately 350 and 900 °C. Finally, this thermal stability indicates that all the compounds can undergo thermal treatment at temperatures up to at least 200 °C for future applications in very hot environments or with significant temperature variations. Although studies regarding the thermal stability of this class of compounds are quite rare, the values for the decomposition temperatures, and consequently their thermal stability, are in accordance with the data reported in the literature.^3,38−40^

To investigate the redox behavior of merocyanines **3**, **6**, and **7**, cyclic voltammetry (CV) measurements in dichloromethane (0.01 M TBAPF_6_) were performed, as shown in Figure 1b. The experimental highest occupied molecular orbital (HOMO) and lowest unoccupied molecular orbital (LUMO) energies of the merocyanine derivatives were determined from the electrochemical data from waves of oxidation and reduction, respectively. The electrochemical and optical bandgaps are summarized in Table 1. The most positive oxidation and the most negative reduction processes were considered, as these processes showed a strong linear correlation with the optical band gap.

**Table 1:** Electrochemical Properties of Merocyanines **3**, **6**, and **7** and Molecular Orbital Energies, where *E*_onset_^oxi^ is the Onset Potential of Oxidation, *E*_onset_^red^ is the Onset Potential of Reduction, *E*_HOMO_ and *E*_LUMO_ are the HOMO and LUMO Energies, Respectively, *E*_gap_^ele^ is the Electrochemical Band Gap, and *E*_gap_^opt^ is the Optical Band Gap

	merocyanine		
parameters	**3**	**6**	**7**
*E*_onset_^oxi,1^ (V) vs Ag/Ag^+^	0.07	0.02	0.06
*E*_onset_^oxi,1^ (V) vs Ag/Ag^+^	0.50	0.42	0.37
*E*_onset_^oxi,3^ (V) vs Ag/Ag^+^	0.81	0.90	0.89
*E*_onset_^red,1^ (V) vs Ag/Ag^+^	–0.53	–0.53	–0.58
*E*_onset_^red,2^ (V) vs Ag/Ag^+^	–1.26	–1.23	–1.22
*E*_HOMO_ (eV)	–4.63	–4.64	–4.59
*E*_LUMO_ (eV)	–4.04	–4.04	–3.99

Merocyanines **6** and **7** have similar structures, differing only in the presence of a sulfur atom in the donor portion, whereas merocyanine **3** has a naphthalene group and a longer alkyl chain at the quaternized nitrogen atom of the heterocyclic moiety. These merocyanines present different processes at different potentials, which correspond to the oxidation of the donor and the reduction of the acceptor moieties, respectively.^41^ In the positive scan, the voltammograms revealed three irreversible processes, and the process with the most positive potential corresponded to the oxidation of the indoline nitrogen, resulting in the formation of a radical cation, as already described in the literature for other derivatives.^42,43^ The other anodic process observed in the voltammogram can be attributed to the double bonding of the π-conjugate system separating the donor from the acceptor, resulting in the formation of a radical cation in the vinyl spacer. This result is similar to others previously reported in the literature.^44,45^ In the negative scan, the voltammograms revealed an irreversible process corresponding to the reduction of the acceptor unit of the merocyanine molecule.^44,45^ A small change in the oxidation potential of the redox processes was observed for merocyanine **6** compared with **3** due to the naphthyl moiety, as was also observed in photophysical studies and theoretical calculations. In this study, all the compounds possessed the same electron withdrawing group, causing a shift to more positive values of the observed potentials than those reported in other works.^46^ Correlation analysis of the electronic data (*E*_onset_^oxi,3^, *E*_gap_^ele^, *E*_gap_^opt^, and *E*_HOMO_) (Figures S15–S17) revealed a good linear correlation between *E*_gap_^opt^ and *E*_gap_^ele^ (*R*^2^ = 0.992). In addition, *E*_onset_^oxi,3^ was linearly correlated with *E*_gap_^opt^ (*R*^2^ = 0.944) and *E*_HOMO_ (DFT) (*R*^2^ = 0.999), suggesting that the electronic properties of the ground state, which are associated with the oxidation potential, affect the photonic properties, thus affecting the band gap of the studied merocyanines.

**Figure 2:**
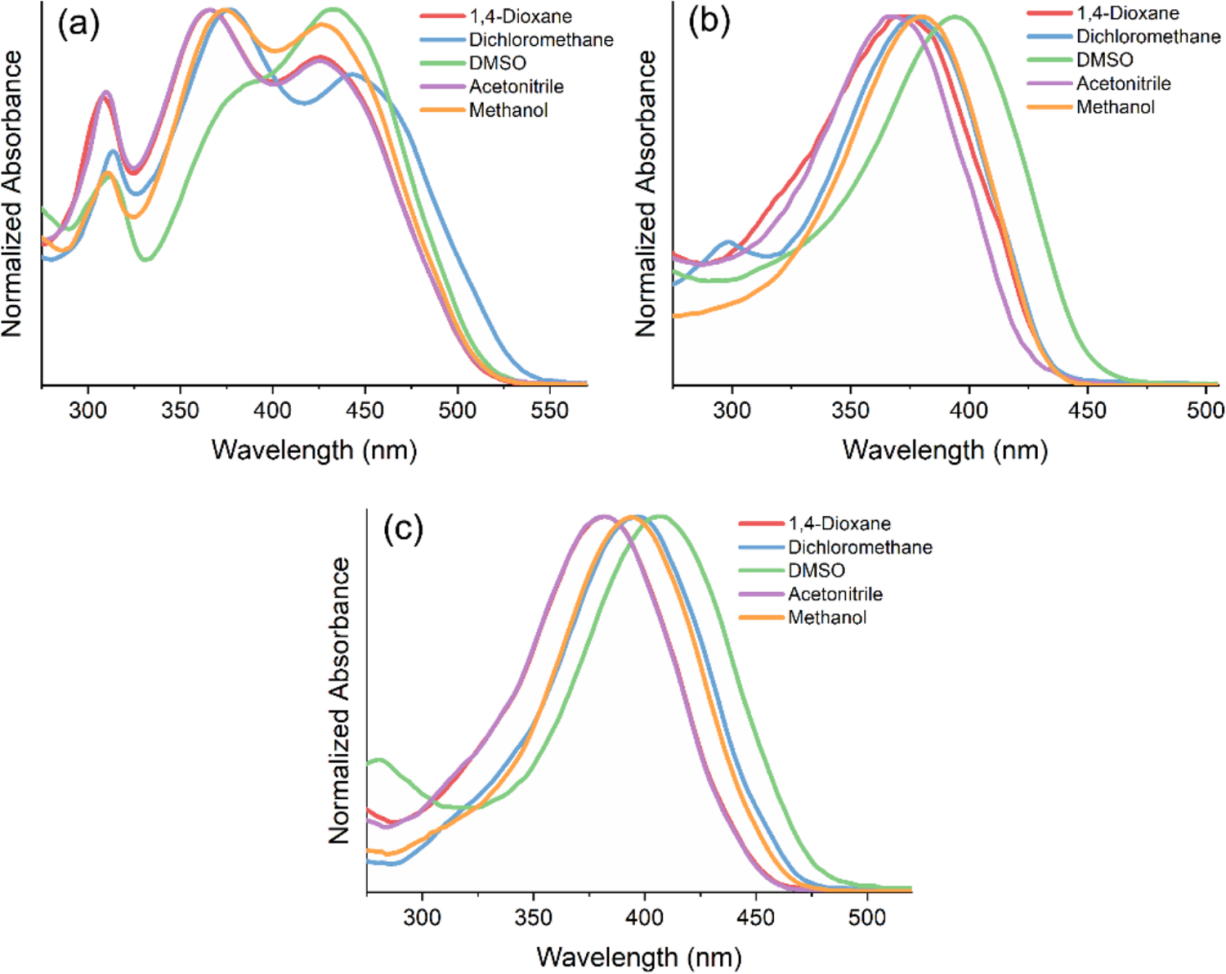
UV–vis absorption spectra of merocyanines (a) **3**, (b) **6**, and (c) **7** in different organic solvents (∼10^–5^ M).

The photophysical properties of the merocyanines were investigated in solution (∼10^–5^ M) using 1,4-dioxane, dichloromethane, DMSO, acetonitrile, and methanol as solvents. The relevant data are summarized in Table 2. Figure 2 shows the ultraviolet–visible (UV–vis) spectra of these derivatives, where absorption maxima between 370 and 442 nm can be observed, depending on their chemical structure. Changes in polarity were found to affect the absorption maxima of merocyanines **3**, **6**, and **7** (Δλ_abs_: 17, 28, and 25 nm, respectively). However, no clear trend in solvatochromism was observed, since any redshift (positive) or blueshift (negative) in the absorption maxima is present, increasing the solvent polarity.^47,48^ This suggests that no CT mechanism is present in the ground state. In addition, merocyanine **3** has two distinct features from those of compounds **6** and **7**. First, the absorption band of merocyanine **3** is red-shifted by approximately 50 nm relative to that of its analogs, a shift attributed to its extended π-conjugation facilitated by the naphthyl moiety. Second, an additional blueshifted absorption band is observed at approximately 375 nm, which is absent in **6** and **7**. This band is believed to correspond to S_0_ → S_2_ electronic transitions. To elucidate the nature of this blueshifted band, the fluorescence emission spectrum of compound **3** was recorded under excitation at 375 nm. The resulting emission spectrum appears in the same region and has the same maximum as that obtained upon excitation at the absorption maximum, suggesting that the compound adheres to Kasha’s rule and that the observed band corresponds to transitions from the ground state to higher-energy electronic levels above S_1_.

**Table 2:** Photophysical Data of Merocyanines **3**, **6**, and **7**^a^

merocyanine	solvent	λ_abs_	ε	*f*	*k*_e_^0^	*τ*^0^	λ_em_	Δλ_ST_	QY
**3**	1,4-dioxane	426	1.87	0.32	1.77	5.64	567	5837	0.45
	dichloromethane	442	1.84	0.32	1.62	6.17	564	4894	0.67
	methanol	428	2.00	0.33	1.80	5.54	565	5665	0.27
	acetonitrile	425	2.14	0.36	1.99	5.03	567	5893	0.50
	dimethylsulfoxide	433	2.62	0.45	2.42	4.14	564	5364	0.20
**6**	1,4-dioxane	370	0.55	0.14	1.05	9.56	470	5751	0.19
	dichloromethane	378	1.57	0.31	2.20	4.56	468	5088	0.11
	methanol	380	1.31	0.24	1.64	6.11	469	4994	0.08
	acetonitrile	366	0.76	0.15	1.15	8.66	457	5441	0.21
	dimethylsulfoxide	394	3.66	0.72	4.67	2.14	474	4284	0.14
**7**	1,4-dioxane	381	2.85	0.62	4.26	2.35	506	6484	0.02
	dichloromethane	397	3.18	0.70	4.44	2.25	518	5884	0.03
	methanol	394	3.17	0.68	4.38	2.28	506	5618	0.02
	acetonitrile	382	2.74	0.61	4.16	2.40	505	6376	0.02
	dimethylsulfoxide	406	3.37	0.71	4.28	2.34	512	5099	0.02

a λ_abs_ is the absorption maxima (nm), ε is the molar extinction coefficient (10^4^ M^–1^·cm^–1^), *f* is the calculated oscillator strength, *k*_e_^0^ is the calculated radiative rate constant (10^8^ s^–1^), τ^0^ is the calculated pure radiative lifetime (ns), λ_em_ and λ_obs_ are the emission and excitation maxima (nm), Δλ_ST_ is the Stokes shift (cm^–1^), and QY is the fluorescence quantum yield (%).

The merocyanines had high molar absorptivities (∼10^4^ M^–1^·cm^–1^), as calculated from the Lambert–Beer law (Table 1). In addition, the Strickler–Berg relations were applied to determine other photophysical parameters, such as the oscillator strength (*f*_e_), theoretical rate constant for emission (*k*_e_^0^), and the pure radiative lifetime τ^0^, defined as 1/*k*_e_^0^.^49,50^ Relatively high values for the oscillator strength could be obtained (0.14–0.72), indicating highly probable electronic transitions. The values from the molar absorptivity coefficient and the calculated radiative rate constant (∼10^8^ s^–1^) corroborate this affirmation. In this sense, the observed absorption maxima could be related to fully spin- and symmetry-allowed ^1^ππ* electronic transitions. Note that τ^0^ represents the intrinsic lifetime of the excited merocyanine molecule, assuming that no radiationless processes are available for it to return to the ground state. In practice, the observed lifetimes are typically shorter than the calculated values because of the presence of competing nonradiative decay pathways.^49^ On the basis of the calculated results (∼10^–9^ s), the studied merocyanines are expected to populate the same excited state.

**Figure 3:**
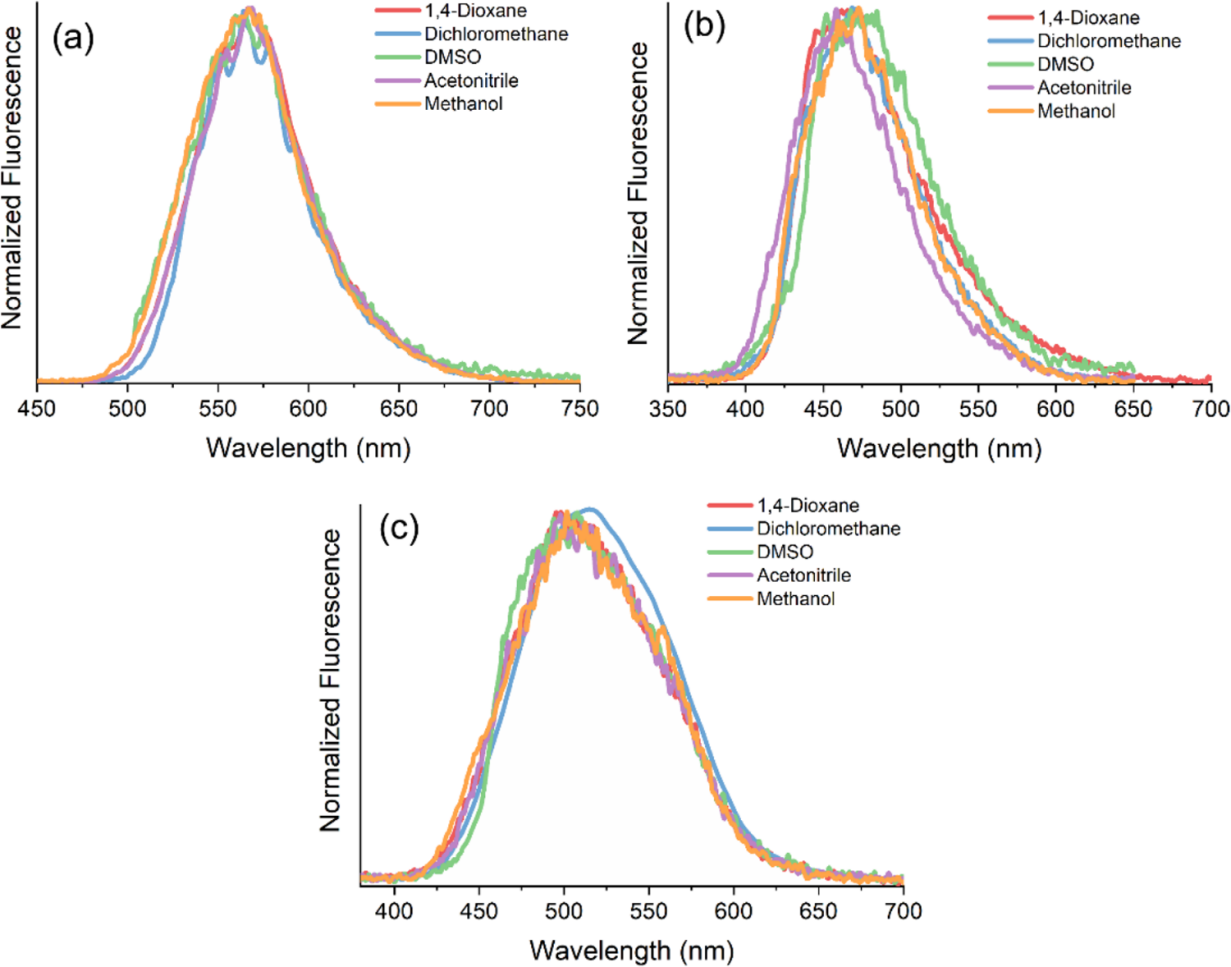
Steady-state fluorescence emission spectra of merocyanines (a) **3**, (b) **6**, and (c) **7** in different organic solvents (∼10^–5^ M).

The merocyanines showed a single fluorescence band located between 420 and 567 nm (**3**:564–567, **6**:420–474, and **7**:505–518 nm), depending on the solvent and the chemical structure of the merocyanines, with moderate Stokes shift values (∼10^3^ cm^–1^). As already observed in the ground state, the observed changes in the location of the emission maxima (Δλ_em_: 3, 17, and 13 nm) show no clear tendency to suggest charge transfer in the excited state (Figure 3). Low fluorescence quantum yields were observed for the studied merocyanines (0.02–0.6%), as already reported in the literature. This is likely due to the rapid photoisomerization characteristic of this class of compounds, which competes with fluorescence.^51,52^ Excitation spectra were also collected using the fluorescence emission maxima as the observation wavelength (Figure S13), revealing a curve profile and maxima location that were comparable to those observed in the UV–vis absorption spectra. These findings suggest that the studied compounds undergo only minimal changes in both their structure and electronic distribution in the excited state.

### Theoretical Calculations

**Figure 4:**
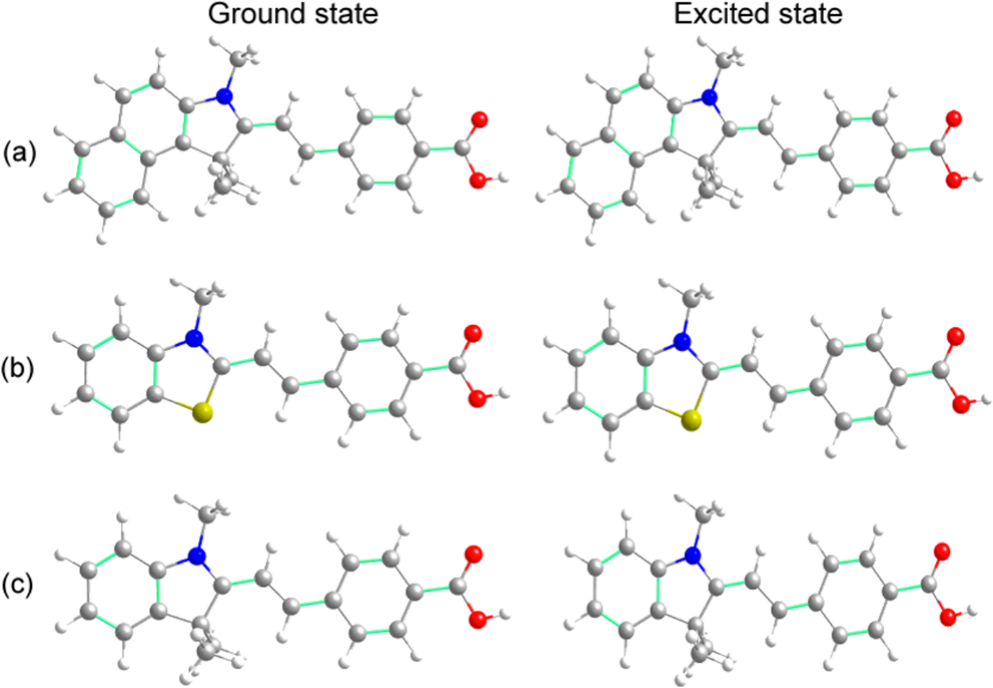
Optimized geometries of the ground and first excited states for merocyanines (a) **3**, (b) **6**, and (c) **7** in acetonitrile obtained at the CAM-B3LYP/cc-pVDZ level of theory.

The use of theoretical calculations in studies involving organic compounds serves multiple key purposes. First, these calculations provide insights into the electronic structure, stability, and geometry. Second, they offer a cost-effective and efficient alternative to experiments, enabling scenario exploration and property prediction. Finally, theoretical predictions guide compound selection and application by predicting properties for comparison with experimental data. In this sense, the theoretical properties of these compounds were evaluated. The merocyanines exhibit planar-optimized structures with C1 symmetry in both the ground and excited states. The rigid architecture of these molecules results in subtle structural changes in the excited state (Figure 4).

Across all three molecules, the absorption wavelength displays minimal variation upon solvent change (Table 3). This observation aligns with experimental findings that suggest that the allowed S_0_ → S_1_ transition lacks charge transfer characteristics.

**Table 3:** Calculated Photophysical Properties of the Merocyanines at the CAM-B3LYP/jun-cc-pVDZ Level of Theory^a^

merocyanine	solvent	λ_abs_	λ_abs_	*f*	μ_GS_	λ_em_	λ_em_	*f*	μ_ES_	λ_ST_
**3**	acetonitrile	417	8	1.177	5.6	553	14	1.228	12.3	5898
	dichloromethane	418	24	1.117	5.4	547	17	1.162	12.1	5642
	1,4-dioxane	419	7	0.921	4.9	531	36	0.936	11.7	5034
**6**	acetonitrile	385	19	1.494	11.5	490	33	1.492	10.5	5566
	dichloromethane	384	6	1.462	11.2	481	13	1.458	10.0	5633
	1,4-dioxane	379	9	1.350	10.2	453	33	1.336	8.8	4310
**7**	acetonitrile	388	6	1.490	9.0	505	0	1.441	11.0	5971
	dichloromethane	387	10	1.447	8.7	497	21	1.393	10.6	5719
	1,4-dioxane	381	0	1.305	7.9	474	32	1.228	9.6	5150

a λ_abs_ and λ_em_ are the absorption and emission wavelengths (nm), λ_abs_ and λ_em_ are the absolute differences between the experimental and theoretical wavelengths, μ_GS_ and μ_ES_ are the dipole moments of the ground state and excited state, respectively, *f* is the oscillator strength and λ_ST_ is the Stokes shift (cm^–1^).

**Figure 5:**
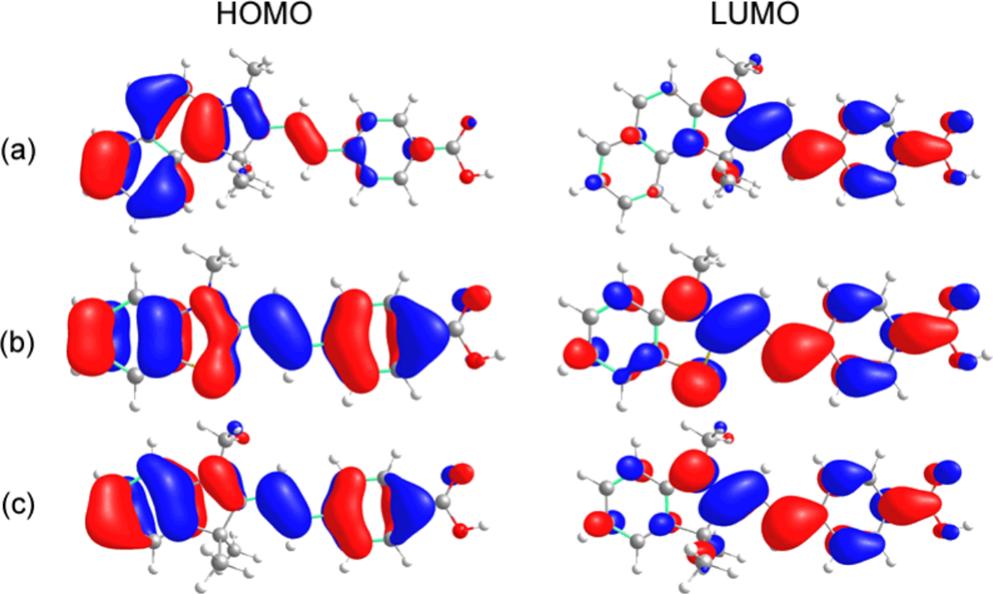
HOMO and LUMO for merocyanines (a) **3**, (b) **6**, and (c) **7** in acetonitrile obtained at the CAM-B3LYP/jun-cc-pVDZ level of theory.

A comparison of merocyanines **3** and **7** revealed an absorption wavelength bathochromic shift. This shift arises from the extra benzene ring in compound **3**, which imparts an energy-increasing influence on the HOMO. As a result, the HOMO in this structure is positioned at approximately −8.1 eV (Table S1), which is approximately 0.6 eV higher than that of merocyanine **7**, which appears at −8.7 eV. The LUMO of compound **3** is located at approximately −3.0 eV, resulting in a HOMO–LUMO gap of approximately 5.1 eV. Similar values were found for the LUMO of compound **7**, leading to a larger HOMO–LUMO gap of 5.6 eV. The introduction of the S atom in compound **6** has a minimal effect on the absorption wavelength compared with that of **7**. The HOMO and LUMO of compound **6** are located at approximately −8.7 and −3.0 eV, respectively, resulting in a HOMO–LUMO gap of approximately 5.7 eV, which is comparable to that of compound **7**. Notably, consistent with the experimental findings, all the resonances of the merocyanines exhibited relatively large Stokes shifts. In the excited state, the dipole moments of compounds **6** and **7** remained relatively stable. In contrast, the dipole moment of merocyanine **3** substantially increased. A bathochromic effect is observed at the emission wavelengths as the dielectric constant of the solvent increases. To ascertain the nature of the transition, an analysis of the HOMO and LUMO orbitals was conducted (Figure 5). Across all three molecules, the HOMO exhibited π character. In merocyanine **3**, the HOMO is predominantly situated on the left side of the molecule, whereas in **6** and **7**, it is evenly distributed throughout. Likewise, the LUMO displayed a π character and demonstrated greater concentration in the middle and right portions of all three molecules. Consequently, the S_0_ → S_1_ transition corresponds to a ^1^ππ* transition.

**Figure 6:**
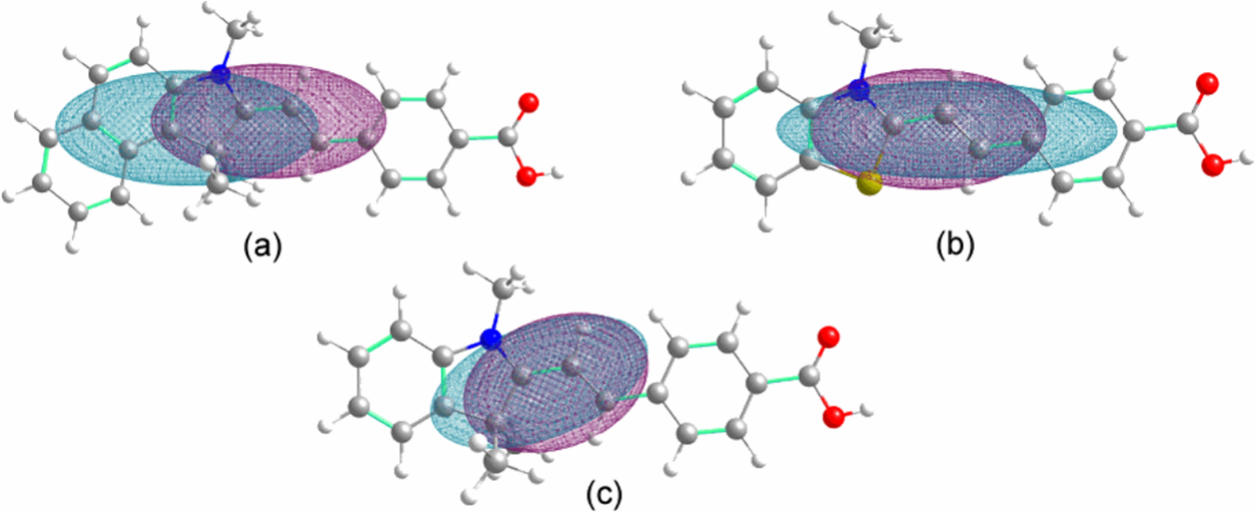
C^–^ (blue) and C^+^ (purple) regions of merocyanines (a) **3**, (b) **6**, and (c) **7** in acetonitrile for the S_0_ → S_1_ transition.

To assess whether the S_0_ → S_1_ transition for **3**, **6**, and **7** has a charge transfer character, the special extent associated with the electronic transition, the DCT index, was evaluated. An examination of the D_CT_ for the three compounds, as summarized in Table 4 and depicted in Figure 6, revealed a distinct pattern. Among the merocyanines, only compound **3** exhibited a significant separation between the regions in which the electronic density increased after excitation (C^+^) and those in which the electronic density decreased (C^–^). However, the negative values of the *t*_*x*_, *t*_*y*_, and *t*_*z*_ indices indicate that these two regions are not effectively distinguished by charge transfer. Furthermore, the modest value of Δσ suggests that C^+^ does not notably differ in diffusion from C^–^. In contrast, for merocyanines **6** and **7**, the *D*_CT_, *t* index, and Δσ do not indicate a charge transfer transition.

**Table 4:** Calculated Values of *D*_CT_ for Merocyanines **3**, **6**, and **7** in Acetonitrile, Dichloromethane, and 1,4-Dioxane

			*t*			
merocyanine	solvent	*D*_CT_	*x*	*y*	*z*	Δσ
**3**	acetonitrile	2.5	–0.77	–1.29	–0.88	0.46
	dichloromethane	2.5	–0.74	–1.30	–0.87	0.40
	1,4-dioxane	2.5	–0.71	–1.35	–0.87	0.23
**6**	acetonitrile	0.5	–2.49	–1.13	–0.82	0.93
	dichloromethane	0.6	–2.37	–1.14	–0.82	0.94
	1,4-dioxane	0.8	–2.14	–1.16	–0.81	0.93
**7**	acetonitrile	0.5	–2.44	–1.46	–0.87	0.93
	dichloromethane	0.4	–2.47	–1.49	–0.87	0.95
	1,4-dioxane	0.3	–2.54	–1.55	–0.86	0.97

### Interaction Studies

**Figure 7:**
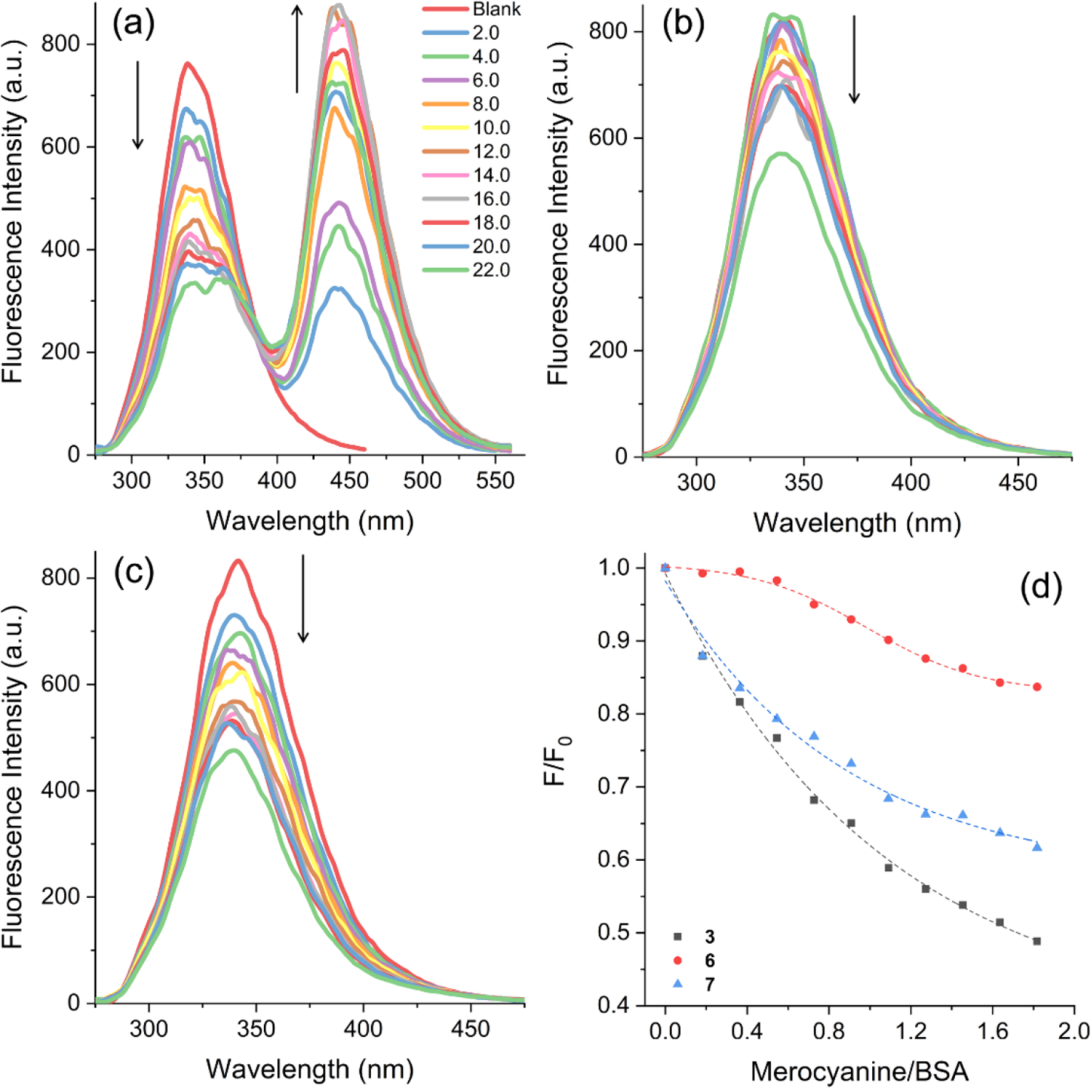
Steady-state fluorescence emission of BSA (11 μM) in PBS at pH 7.4 in the absence and presence of different amounts (0–22 μM) of merocyanines (a) **3**, (b) **6**, and (c) **7**. (d) Fluorescence emission intensities of BSA relative to the concentration of merocyanines in PBS at room temperature.

A BSA suppression study was performed by keeping the BSA concentration constant (11.0 μM in PBS, pH 7.2) followed by titration with different amounts of a previously prepared solution of merocyanine (0–22.0 μM in dimethylformamide). First, the absorption spectra of these compounds demonstrated that as the concentration increased, there was a distinct improvement in the absorption region related to the merocyanines, as expected (Figure S14). Fluorescence quenching assays were conducted to investigate the interactions between the merocyanines and BSA, as illustrated in Figure 7. The fluorescence spectrum of BSA presented an emission maximum of 334 nm. However, upon the gradual addition of the merocyanines, a reduction in fluorescence intensity was observed without any significant redshift in either scenario. These observations indicate that the studied compounds likely interact in proximity to the tryptophan residue (Trp-214 for HSA and Trp-134 or Trp-212 for BSA). Additionally, the introduction of these compounds did not disrupt the microenvironment around the tryptophan binding site, as indicated by previous studies.^53^ In general, the merocyanines demonstrated substantial interactions with BSA, resulting in significant suppression of fluorescence ranging from 32 to 56% (Table 5). Titration with merocyanine **3** resulted in a concomitant increase in the emission intensity at 475 nm, increasing the compound concentration, as expected. The excitation of BSA at 279 nm also excites the compound because its absorption is significantly broader than that of the other studied compounds.

**Table 5:** Results Obtained from the Interaction Study of the Merocyanines with BSA^a^

merocyanine	*F*_0_/*F*	*Q* (%)	*K*_SV_ (L·mol^–1^)	*K*_q_ (L·mol^–1^·s^–1^)	log[(*F*_0_ – *F*)/*F*]	*K*_a_ (L·mol^–1^)	*n*
**3**	1.012 + 5.34 × 10^4^[*Q*] (0.9879)	56	5.34 × 10^4^	8.81 × 10^12^	4.766 + 1.005log[*Q*] (0.9920)	5.83 × 10^4^	1.005
**6**	0.9627 + 1.20 × 10^4^[*Q*] (0.9743)	32	1.20 × 10^4^	1.98 × 10^12^			
**7**	1.095 + 2.72 × 10^4^[*Q*] (0.9817)	42	2.72 × 10^4^	4.49 × 10^12^	3.350 + 0.760log[*Q*] (0.9827)	2.24 × 10^4^	0.760

a *Q* (%) = *F*_0_ – *F*/*F*_0_ × 100.

**Figure 8:**
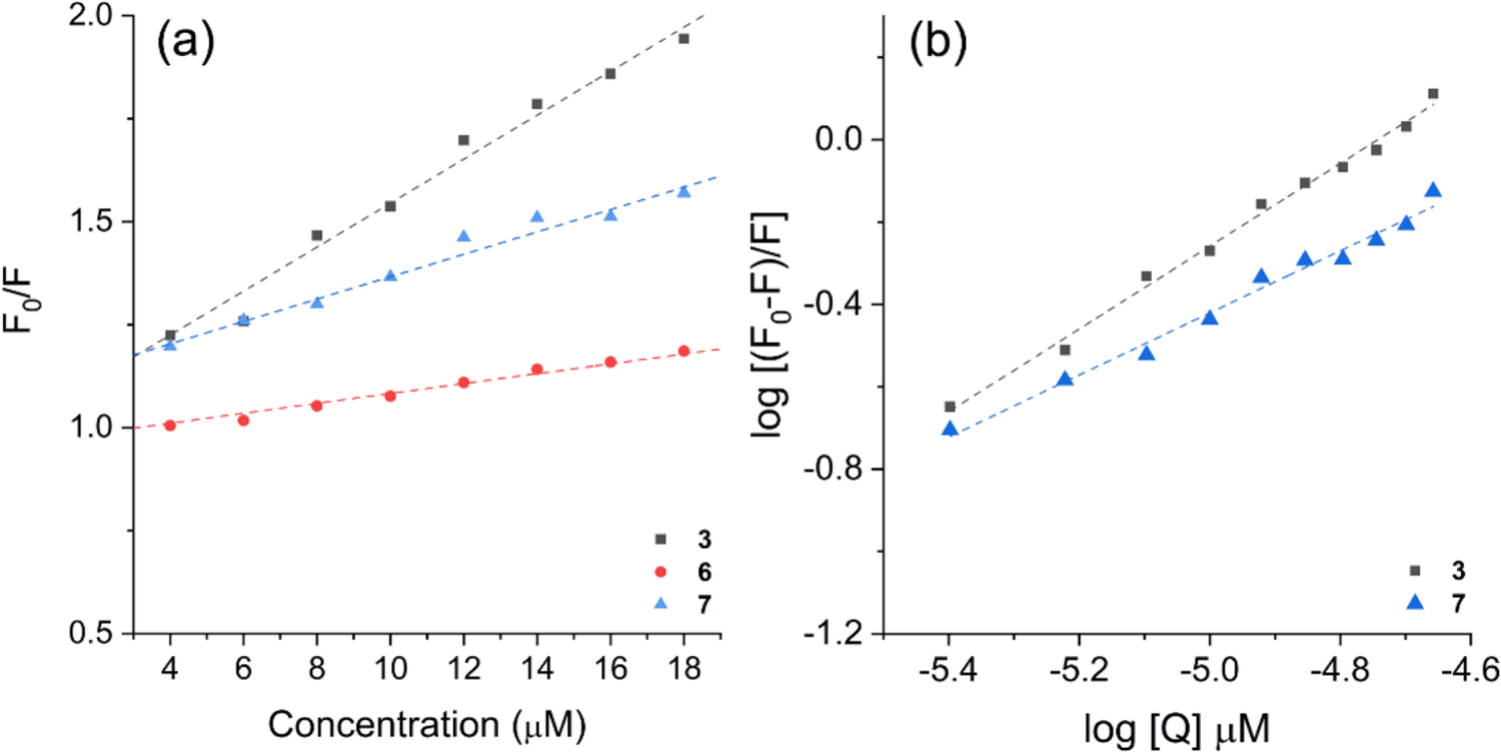
(a) Stern–Volmer curves of BSA quenching in the presence of merocyanines **3**, **6**, and **7** and (b) double logarithm plots used to calculate the binding constant (*K*_a_) and the number of binding sites (*n*) with BSA.

Furthermore, the plot depicting the relative fluorescence intensity of BSA (*F*/*F*_0_) against the merocyanine/BSA ratio (Figure 7d) indicates that these compounds have distinct interactions with BSA. Merocyanine **3** showed the most significant reduction in BSA emission at lower dye/protein ratios compared with the other analogs under study. To explore the underlying mechanism of BSA fluorescence quenching and to derive the associated parameters for this interaction, Stern–Volmer curves were generated via eq 1. Here, *F*_0_ represents the fluorescence intensity of the pure BSA solution, *F* represents the fluorescence intensity of BSA in the presence of the merocyanines (quenchers), *K*_q_ represents the bimolecular quenching constant, which is indicative of the quenching efficiency, and τ^0^ represents the lifetime of the fluorophores in the absence of the quencher (6.06 ns).^54^ The concentration of merocyanine is represented as [*Q*], and the Stern–Volmer quenching constant is denoted as *K*_SV_.^55^ A graphical representation of the Stern–Volmer plots is shown in Figure 8a, and the relevant findings from this investigation are summarized in Table 5.
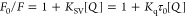
1

The merocyanines presented *K*_SV_ values of approximately 10^4^ L·mol^–1^, with compound **3** revealing the highest suppression constant among them. This outcome signifies that this molecule is more effective at quenching BSA than the other studied merocyanines. The bimolecular quenching constant, *K*_q_, was also obtained, with a value of approximately 10^12^ L·mol^–1^·s^–1^. These values significantly surpass the upper limits of the diffusional mechanism (2.0 × 10^10^ M^–1^·s^–1^), suggesting that the observed fluorescence quenching arises from a static mechanism.^53^ In this scenario, merocyanine/BSA complex formation occurs in the ground state.

In addition, given that the concentration of the merocyanines (quenchers) is considered equal to their total quantity within the solution and given the direct correlation between fluorescence intensity (*F*) and the concentration of the nonemissive complex merocyanine/BSA, it is possible to determine the association binding constant (*K*_a_) and the number of binding sites (*n*) via the Scatchard relation (eq 2).^56^ In this equation, *F*_0_ and *F* denote the fluorescence intensities before and after the addition of the quencher, respectively, and [*Q*] represents the concentration of the merocyanine. As shown in Figure 8b, double logarithm plots illustrating the relationship between the BSA fluorescence intensity and the concentration of merocyanines **3** and **7** revealed a linear correlation. Notably, only these two compounds exhibited a linear fit within the studied observation window.
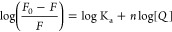
2

These results indicate that the studied merocyanines significantly interact with BSA, as evidenced by their high binding constant of approximately 10^4^ L·mol^–1^. Additionally, the number of binding sites was approximately 1, suggesting that only a single merocyanine is accommodated within the protein cavity.

Molecular docking has become an increasingly important tool for drug discovery, suggesting insights that improve the understanding of the molecular behavior between a potential drug and protein.^57^ Thus, to offer an atomic-level explanation of the binding capacity of the studied merocyanines to BSA by suggesting the main intermolecular forces in the binding process, as well as the most likely binding site, i.e., sites I, II, or III (subdomains IIA, IIIA, and IB, respectively), in silico studies via molecular docking calculations were carried out. As described in the experimental fluorescence quenching results, the addition of the merocyanines at different concentrations decreased the fluorescence emission of BSA, so it can be concluded that these ligands bind next to a fluorophore of albumin. BSA has two main fluorophoric groups that possess the highest intrinsic fluorescence: the Trp-134 residue, located on the surface of the albumin structure in subdomain IB (site III), and the Trp-212 residue, located within a hydrophobic binding pocket in subdomain IIA (site I).^58,59^ In addition, subdomain IIIA, also known as site II, was determined to be a promising binding site for different compounds, despite it not having a Trp residue. In this sense, the ligands bound in this region could interact with the Trp-212 residue through energy transfer.^60^Table 6 shows the docking score values (dimensionless) for BSA:merocyanines inside the three most likely binding sites. Since site I showed the highest docking score for the three ligands under study, e.g., for BSA:3, the docking scores were 69.21, 60.16, and 58.20 for sites I, II, and III, respectively, in silico studies suggested that subdomain IIA, where the Trp-212 residue can be found, was the most promising binding site. According to the literature, commercial merocyanine 540 and the novel merocyanine dye 4-[2,3-dimethyl-3(2-methylamino-phenyl)-but-1-enyl]-benzene-1,2-diol bind preferentially at site I,^25,61^ corroborating the in silico results. Interestingly, when the docking scores of the three ligands at site I were compared, compound 3 presented the highest value, suggesting that it has a greater affinity for BSA than the other ligands do, which is in further accordance with the decrease in the experimental *K*_a_ value for 3 > 6 > 7. Furthermore, the in silico results suggested one main binding site, in accordance with the experimental results (*n* ∼ 1.0).

**Table 6:** Molecular Docking Score Values (Dimensionless) for the Interaction of BSA:Merocyanines

merocyanine	site I	site II	site III
**3**	69.21	60.16	58.20
**6**	66.90	59.19	57.86
**7**	64.89	56.03	57.45

**Figure 9:**
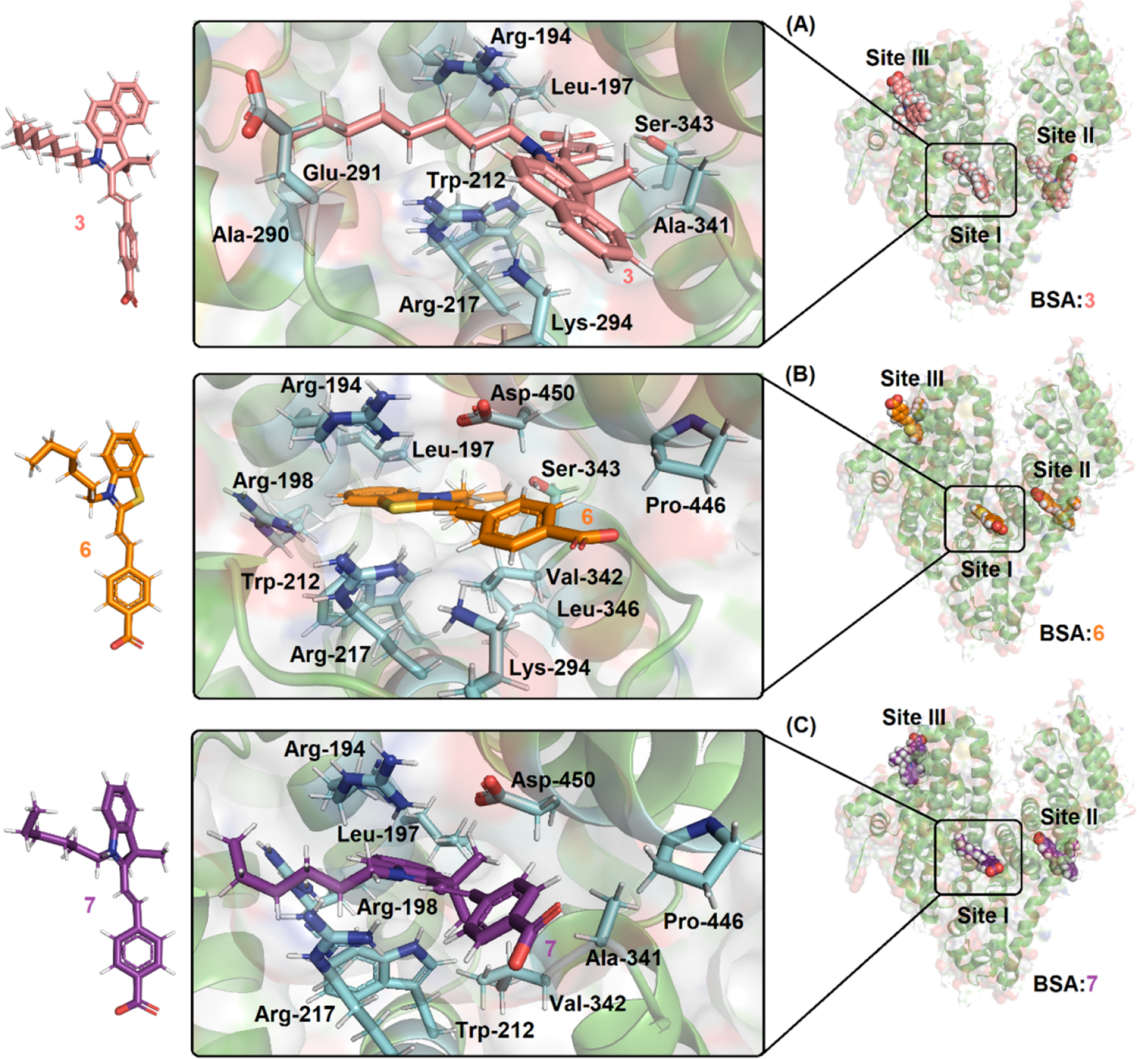
Best docking poses of the interactions of (A) BSA:**3**, (B) BSA:**6**, and (C) BSA:**7** at pH = 7.4. The selected amino acid residues **3**, **6**, and **7** are shown in stick representation in cyan, beige, orange, and purple, respectively. The elements are as follows: hydrogen, white; oxygen, red; nitrogen, dark blue; and sulfur, yellow.

Figure 9 shows the best docking pose for the interaction between BSA:5–7 at site I, and Table 7 shows the main amino acid residues that interact with the ligands. According to the molecular docking results, merocyanine 3 is buried inside the protein pocket in a different pose than 6 and 7, which showed that the aromatic carboxylic acid moiety is more available to the aqueous medium, providing insights into the difference in the binding capacity of 3 compared with the other two molecules. Hydrogen bonding and van der Waals interactions were suggested as the main intermolecular forces responsible for the association of BSA:merocyanines, whereas interactions via *t*-stacking were detected only for BSA:6 and BSA:7. For example, the hydrogen from Trp-212 (−NH) and Ser-343 (−OH) is a potential donor for hydrogen bonding with the carboxylic group of 3 structures within distances of 3.00 and 2.10 Å, respectively, whereas the amino acids Arg-194, Leu-197, Arg-217, Ala-290, Glu-291, Lys-294, and Ala-341 interact with 3 via van der Waals forces within distances of 3.50, 2.20, 1.90, 3.50, 1.90, 2.70, and 2.70 Å, respectively (Table 7).

**Table 7:** Molecular Docking Results for the Interaction of BSA with the Merocyanines at Site I

merocyanine	amino acid residues	interaction	distance (Å)
**3**	Arg-194	van der Waals	3.50
	Leu-197	van der Waals	2.20
	Trp-212	hydrogen bonding	3.00
	Arg-217	van der Waals	1.90
	Ala-290	van der Waals	3.50
	Glu-291	van der Waals	1.90
	Lys-294	van der Waals	2.70
	Ala-341	van der Waals	2.70
	Ser-343	hydrogen bonding	2.10
**6**	Arg-194	hydrogen bonding	2.60
	Leu-197	van der Waals	2.00
	Arg-198	van der Waals	2.40
	Trp-212	*t*-stacking	2.80
	Arg-217	van der Waals	3.10
	Lys-294	van der Waals	2.70
	Val-342	van der Waals	2.10
	Ser-343	van der Waals	3.20
	Leu-346	van der Waals	2.10
	Pro-446	van der Waals	2.60
	Asp-450	van der Waals	1.80
**7**	Arg-194	van der Waals	1.70
	Leu-197	van der Waals	1.80
	Arg-198	van der Waals	2.70
	Trp-212	*t*-stacking	3.20
	Arg-217	van der Waals	3.10
	Ala-341	van der Waals	2.70
	Val-342	van der Waals	3.20
	Pro-446	van der Waals	3.10
	Asp-450	van der Waals	3.10

## Conclusions

In summary, we report here the synthesis of merocyanine dyes by the Knoevenagel condensation reaction with good yields. This reaction was carried out between an aldehyde and indolium/benzoindolium and benzothiazolium moieties in a basic environment. The studied merocyanines were thermally stable, with initial decomposition temperatures of approximately 220 °C. In general, we observed a good linear correlation between the electrochemical and electronic data, which was supported by the DFT data. The merocyanines presented absorption between 366 and 442 nm, related to electronic transitions with a ^1^ππ* character, and fluorescent emission in the blue-to-green region, characterized by a relatively large Stokes shift. The merocyanines exhibited BSA suppression effects, as demonstrated by high Stern–Volmer (∼10^4^ M^–1^) and bimolecular suppression (∼10^12^ M^–1^·s^–1^) constants. Steady-state experiments confirmed that the suppression mechanism was static. Molecular docking revealed the following possible binding sites: subdomains IIA (site I), IIIA (site II), and IB (site III). Experimental fluorescence quenching aligned with the ligands binding near the fluorophores of BSA. Trp-134 (site III) and Trp-212 (site I) are key fluorophoric residues. Subdomain IIIA also exhibited potential for binding through energy transfer. The docking scores indicated that site I was most favorable, which was consistent with previous findings. Notably, merocyanine **3** displayed the highest docking score and affinity for BSA, corroborating the experimental results. This study established a primary binding site that matches experimental observations.

## Experimental Section

### General Information

The reagents 2,3,3-trimethyl-3*H*-indole, 2-methylbenzothiazole, 1-iodooctane, 1-iodohexane, *N*,*N*-dimethylformamide, methanol, ammonium acetate, and 4-formylbenzoic acid were purchased from Sigma–Aldrich. The solvents and reagents were used as received or purified via standard procedures.^62^ Silica Gel 60 F254 was used for thin-layer chromatography, and silica Gel 60 Å (70–230 mesh) was used for column chromatography. Hydrogen and carbon nuclear magnetic resonance (^1^H and ^13^C NMR) data were recorded in DMSO-*d*_6_ or MeOD-*d*_4_ at 400 and 100 MHz, respectively. The chemical shifts (δ) are reported in parts per million (ppm) relative to TMS (0.00 ppm), and the coupling constants *J* are reported in hertz (Hz). The compounds were also characterized via Fourier transform infrared (FTIR) spectroscopy (750–4000 cm^–1^) recorded by a Shimadzu IR Prestige-21 spectrophotometer with a resolution of 4.0 cm^–1^ using KBr pellets. High-resolution mass spectra (HRMS) were recorded on a Micromass Q-ToF spectrometer via electrospray ionization (ESI). The thermal stability of the compounds was investigated via thermogravimetric analysis (TGA). TGA measurements were performed via a TG 50 Shimadzu thermal analyzer with a heating rate of 10 °C·min^–1^ from 25 to 900 °C under a nitrogen gas atmosphere. Differential scanning calorimetry was performed on a DSC-50 instrument using a N_2_ atmosphere with a flow rate of 100 mL·min^–1^ and a heating rate of 10 °C·min^–1^. UV–vis absorption spectra in solution were obtained on a Shimadzu UV-2450 spectrophotometer at a dye concentration of 10^–5^ M. Steady-state fluorescence spectra were taken using a Shimadzu spectrofluorometer model RF-5301PC. The maximum absorption wavelength was used as the excitation radiation for the emission spectra. Slits of 5.0 nm/5.0 nm were used for emission/excitation, respectively. The quantum yield of fluorescence was measured at 25 °C using spectroscopic grade solvents within solutions with an absorbance intensity lower than 0.1 (optical dilution method). Coumarin 153 (ethanol solution) was used as the quantum yield standard for merocyanine **3**, and coumarin 30 (ethanol solution) was used for merocyanines **6** and **7**.^63^

### Synthesis

In general, benzoindolium, benzothiazolium, and indolium coupling compounds **2**, **4**, or **5**, respectively (0.5 mmol), and CH_3_COONH_4_ (38 mg, 0.5 mmol) were dissolved in methanol (20 mL), and the mixture was stirred for 10 min at 25 °C. Then, 4-formylbenzoic acid (**1**) (75 mg, 0.5 mmol) was added to the reaction mixture and heated under reflux for 12 h. After the solvent was evaporated, the solid was collected, washed with dichloromethane (3 × 50 mL), and vacuum-dried to afford a colored solid. The original spectra from the spectroscopic characterization can be found in the Supporting Information (Figures S1–S12).

#### Merocyanine **3**

Yield: 65% (orange solid). Melting point: 210–212 °C. FTIR (ν_max_/cm^–1^): 3392, 2930, 2852, 1702, 1593, 1280. ^1^H NMR (DMSO-*d*_6_, 400 MHz): 8.63 (d, 1H, *J* = 16.0 Hz), 8.47 (d, 2H, *J* = 8.0 Hz), 8.38 (d, 2H, *J* = 8.0 Hz), 8.33 (d, 2H, *J* = 8.0 Hz), 8.25 (d, 1H, *J* = 8.0 Hz), 8.28 (d, H, *J* = 16.0 Hz), 8.12 (d, 2H, *J* = 8.0 Hz), 7.82 (m, 3H), 4.91 (t, 2H, *J* = 8.0 Hz), 2.06 (s, 6H), 1.93 (m, 2H), 1.45 (m, 2H), 1.33 (m, 2H), 1.19 (m, 6H), 0.78 (t, 3H, *J* = 6.0 Hz). ^13^C NMR (DMSO-*d*_6_, 100 MHz): 182.9, 167.1, 151.1, 139.7, 138.7, 134.3, 133.9, 130.8, 130.2, 129.0, 128.1, 127.1, 123.8, 114.8, 114.0, 54.6, 47.6, 31.6, 28.9, 25.8, 22.4, 14.36. HRMS (ESI) calcd for C_31_H_36_NO_2_ (M)^+^ requires 454.2741, found 454.2718.

#### Merocyanine **6**

Yield: 72% (yellow solid). Melting point: 169–172 °C. FTIR (ν_max_/cm^–1^): 3423, 2923, 2860, 1710, 1608. ^1^H NMR (MeOD-*d*_4_, 400 MHz): 8.63 (d, 1H, *J* = 16.0 Hz), 8.47 (d, 2H, *J* = 8.0 Hz), 8.38 (d, 2H, *J* = 8.0 Hz), 8.33 (d, 2H, *J* = 8.0 Hz), 8.25 (d, 1H, *J* = 8.0 Hz), 8.28 (d, H, *J* = 16.0 Hz), 8.12 (d, 2H, *J* = 8.0 Hz), 7.82 (m, 3H), 4.91 (t, 2H, *J* = 8.0 Hz), 2.06 (s, 6H), 1.93 (m, 2H), 1.45 (m, 2H), 1.33 (m, 2H), 1.19 (m, 6H), 0.78 (t, 3H, *J* = 6.0 Hz). ^13^C NMR (MeOD-*d*_4_, 100 MHz): 171.8, 169.2, 148.4, 141.5, 136.8, 129.9, 129.1, 128.8, 123.9, 116.6, 49.4, 31.0, 28.9, 25.8, 22.1, 12.9. HRMS (ESI) calcd for C_22_H_24_NO_2_S (M)^+^ requires 366.1522, found 366.1502.

#### Merocyanine **7**

Yield: 60% (yellow solid). Melting point: 214–215 °C. FTIR (ν_max_/cm^–1^): 3400, 2930, 2860, 1702, 1600, 1287. ^1^H NMR (DMSO-*d*_6_, 400 MHz): 8.53 (d, 1H, *J* = 16.0 Hz), 8.36 (d, 2H, *J* = 8.0 Hz), 8.10 (d, 2H, *J* = 8.0 Hz), 8.02 (m, 1H), 7.94 (m, 1H), 7.83 (d, 1H, *J* = 16.0 Hz), 7.67 (m, 2H), 4.78 (t, 2H, *J* = 6.0 Hz), 1.84 (s, 6H), 1.44 (m, 2H), 1.27 (m, 6H), 0.83 (t, 3H, *J* = 6.0 Hz). ^13^C NMR (DMSO-*d*_6_, 100 MHz): 182.4, 167.1, 152.3, 144.6, 141.1, 138.5, 134.5, 130.9, 130.2, 123.6, 116.2, 115.2, 53.1, 47.5, 31.2, 28.9, 25.9, 22.3, 14.2. HRMS (ESI) calcd for C_25_H_30_NO_2_ (M)^+^ requires 376.2271, found 376.2262.

### BSA Binding Study

A bovine serum albumin (BSA) suppression study was performed while keeping the BSA concentration constant (11.0 μM in PBS, pH 7.2), and a previously prepared solution (0–22.0 μM in dimethylformamide) of the merocyanines was added. The final solution was allowed to rest for 1 h. Fluorescence spectra were obtained at 25 °C under excitation at 279 nm. Slits of 5.0/5.0 nm were used for emission/excitation, respectively. The BSA association study was performed by keeping the merocyanine concentration constant (2.0 μM in dimethylformamide), and a previously prepared BSA solution (0–12.0 μM in PBS, pH 7.2) was added. The final solution was allowed to rest for 1 h. The fluorescence spectra of merocyanines **3**, **6**, and **7** were obtained at 25 °C and under excitation at 430, 380, and 392 nm, respectively. Slits of 5.0 nm/5.0 nm were used for emission/excitation, respectively.

### Molecular Docking

The BSA crystallographic structure was obtained from the Protein Data Bank (PDB), with access code 4F5S.^58^ The chemical structure of the merocyanines was built and minimized in terms of energy by density functional theory (DFT), which is available in Spartan’18 software (Wave function, Inc., Irvine, CA).^64^ Molecular docking calculations were performed with GOLD 5.7 software (Cambridge Crystallographic Data Centre, Cambridge, CB2 1EZ, U.K.).^65^ Hydrogen atoms were added to the protein following tautomeric states, and ionization data were inferred via GOLD 5.7 software. For the molecular docking, calculations were defined as a 10 Å radius around the three main binding pockets of the albumin (subdomains IIA, IIIA, and IB—known as sites I, II, and III, respectively).^66,67^ The standard *ChemPLP* function was used owing to the best results obtained in previous work for small heterocyclic compounds.^59,66−^ The figures of the best docking pose were generated with PyMOL Delano Scientific LLC software.^69^

### Electrochemical Characterization

Cyclic voltammograms (CVs) of merocyanines **3**, **6**, and **7** were measured with a three-electrode electrochemical cell with a PalmSens3 potentiostat/galvanostat (Palm Instruments BV). A glassy carbon disk, a platinum wire, and Ag/Ag^+^ (0.01 M AgNO_3_ in acetonitrile) were employed as the working electrode, counter electrode, and reference electrode, respectively. All CV scans were performed at a scan rate of 100 mV·s^–1^. The supporting electrolyte was a nonaqueous CH_2_Cl_2_ solution containing 0.01 M tetrabutylammonium hexafluorophosphate (TBAPF_6_), which was degassed with argon to remove dissolved oxygen. All potential measurements were calibrated (versus NHE) using a ferrocene/ferrocenium redox couple (0.27 vs Ag/Ag^+^), which was used as the internal standard, as recommended by the IUPAC. Potentials measured vs Fc^+^/Fc were recalculated to absolute potentials.^46^ The following equations were used to calculate the approximate HOMO and LUMO energies: *E*_HOMO_ = −(*E*_onset_^oxi^ + 4.44) eV and *E*_LUMO_ = −(*E*_onset_^red^ + 4.44) eV; *E*_onset_^oxi^ and *E*_onset_^red^ are the onset potentials of oxidation and reduction, respectively. The difference between the HOMO and LUMO energy levels gives the electrochemical bandgap, *E*_gap_^ele^.

### Theoretical Calculations

The equilibrium structures of the ground and excited states were optimized at the CAM-B3LYP/cc-pVDZ level of theory, and both were characterized by the absence of imaginary frequencies. Excitation energies were obtained at the CAM-B3LYP/jun-cc-pVDZ level of theory considering the first ten excited states. CAM-B3LYP was chosen because long-range corrections are necessary.^70^ The jun-cc-pVDZ basis set was chosen because diffuse orbitals are necessary to adequately describe excited states.^71,72^ Both density functional theory (DFT) and time-dependent density functional theory (TD-DFT) calculations were carried out via the Gaussian16 package.^73^ Acetonitrile, dichloromethane, and 1,4-dioxane were implicitly simulated via the polarizable continuum model (PCM).^74^ Dipole moments were evaluated by charging from the electrostatic potential via the grid-base (ChelpG) method.^75^ To evaluate the charge transfer character of the electronic transition, the electronic density difference was analyzed with Multiwfn software.^76,77^ Geometry, increment (C^+^), and depletion (C^–^) region figures were plotted with CHEMCRAFT software.^78^

## Supporting Information Available

Original spectra from spectroscopic characterization and additional photophysical data (PDF)
