# Supplementary material for: Synthesis, Characterization, and BSA Binding Properties of Carboxylated Merocyanine-Based Fluorophores
Source: ACS Omega. 2024 Nov 26;9(49):48697–710. doi: 10.1021/acsomega.4c07997 (PMC11635679; doi:10.1021/acsomega.4c07997)
Supplement: Supplementary file 1 — ao4c07997_si_001.pdf [file ao4c07997_si_001.pdf]

Synthesis, characterization and BSA binding properties of carboxylated merocyanine-based fluorophores

Rodrigo C. Duarte,[a] Rodrigo Cercená,[a] Bruno B. de Araujo,[b] Otávio A. Chaves,[c] Paulo F. B. Gonçalves,[b] Eduardo Zapp,[d] Fabiano S. Santos,[b] Fabiano S. Rodembusch,[b]* Alexandre G. Dal-Bó[a]*

[a]Universidade do Extremo Sul Catarinense (UNESC). Av. Universitária, 1105. CEP 88806-000. Criciúma-SC, Brazil. E-mail: adalbo@unesc.net

[b]Instituto de Química, Universidade Federal do Rio Grande do Sul (UFRGS), Porto Alegre CEP 91501-970, Brazil. E-mail: rodembusch@iq.ufrgs.br

[c]CQC-IMS, Department of Chemistry, University of Coimbra, Rua Larga s/n, 3004-535, Coimbra, Portugal

[d]Universidade Federal de Santa Catarina. Departamento de Ciências Exatas e Educação (CEE). Blumenau, 89036-004, Brazil

Supporting Information

Spectroscopic characterization

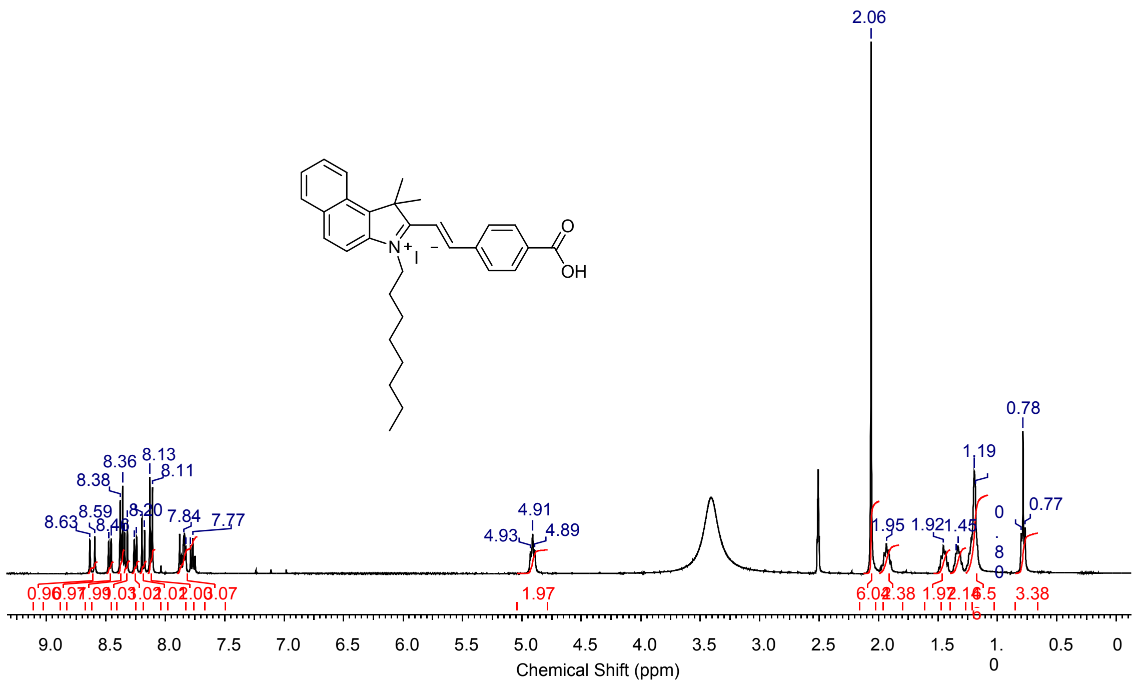

Figure S1. ^{1}H NMR spectrum (DMSO-d_6, 400 MHz) of merocyanine **3**.

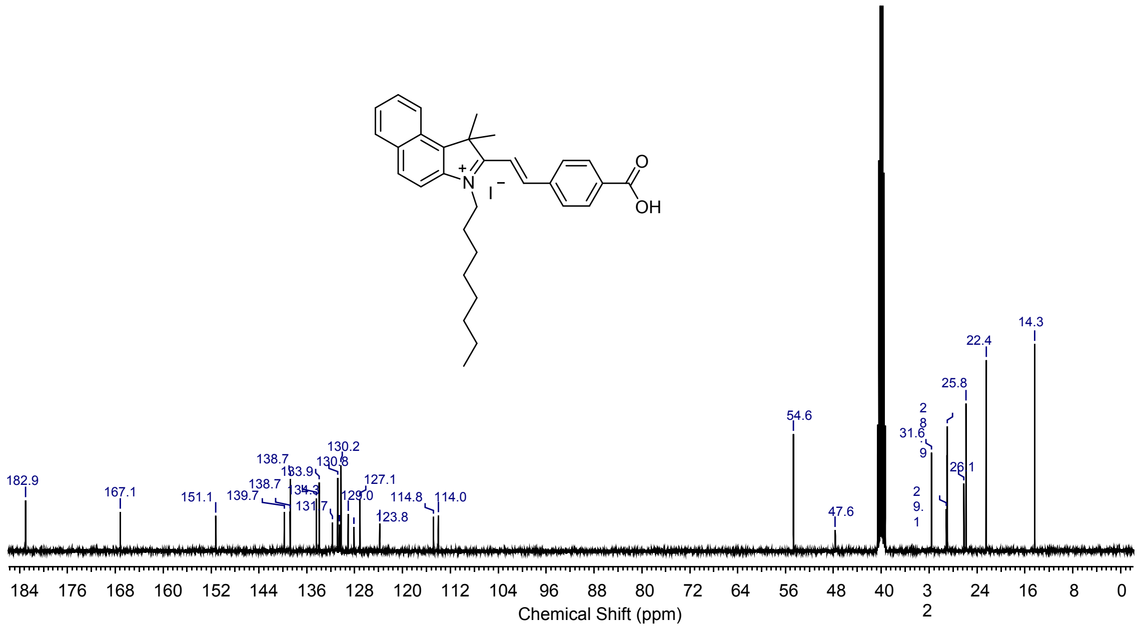

Figure S2. ^{13}C NMR spectrum (DMSO-d_6, 100 MHz) of merocyanine **3**.

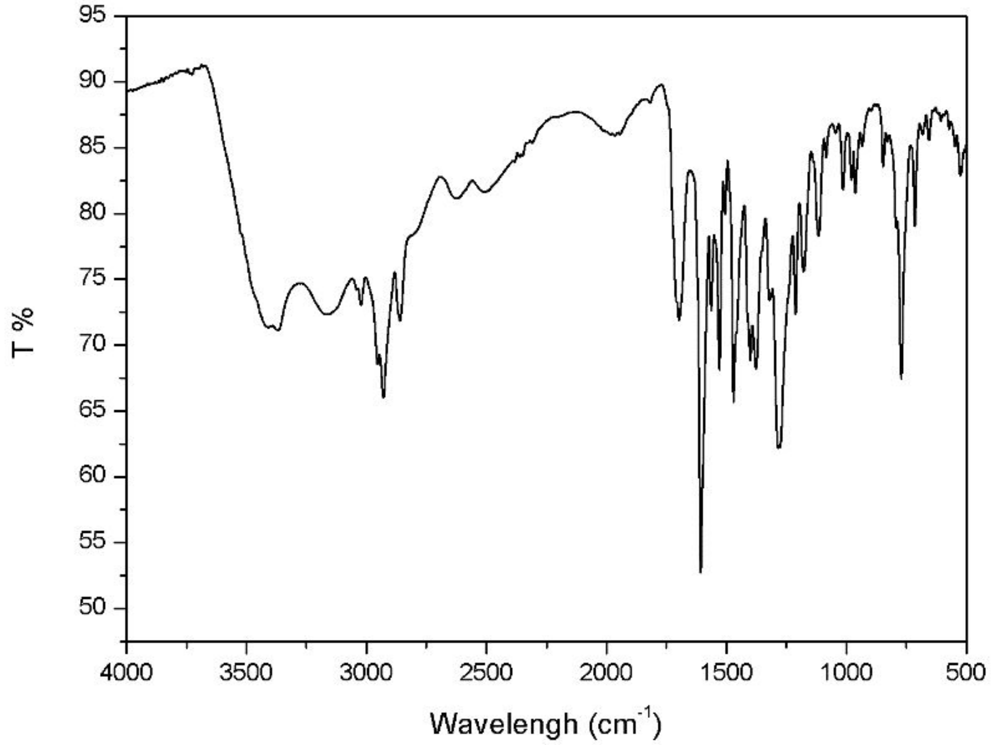

Figure S3. FTIR spectrum of merocyanine **3**.

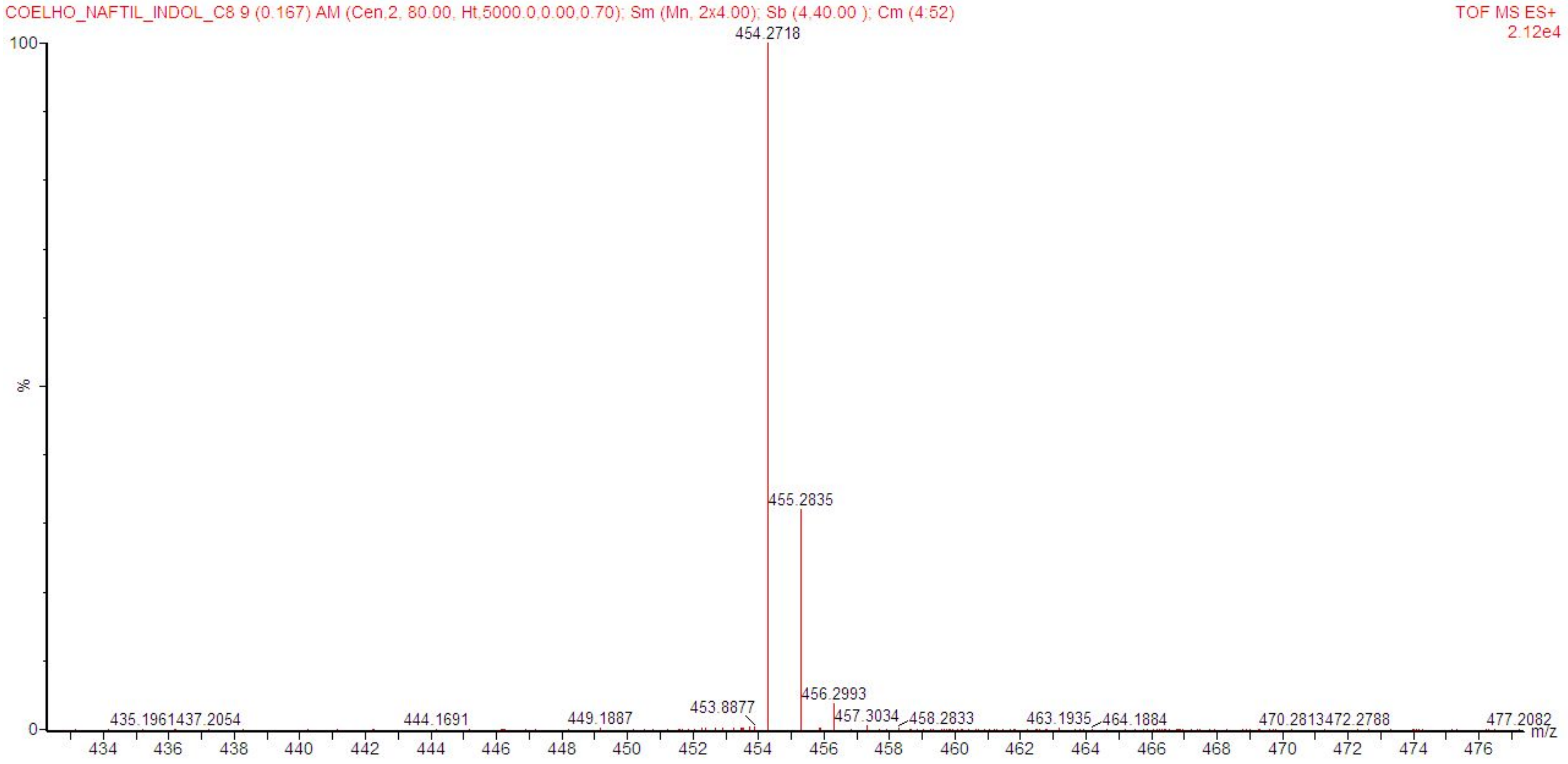

Figure S4. ESI-MS analysis of merocyanine **3**.

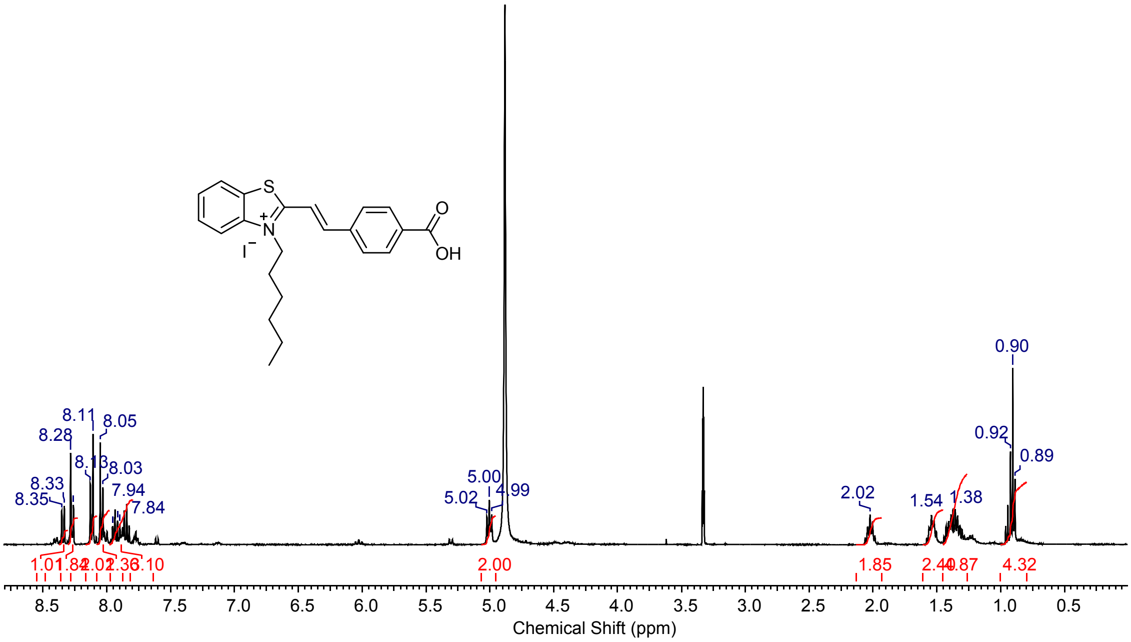

Figure S5. ^{1}H NMR spectrum (MeOD-d_4, 400 MHz) of merocyanine **6**.

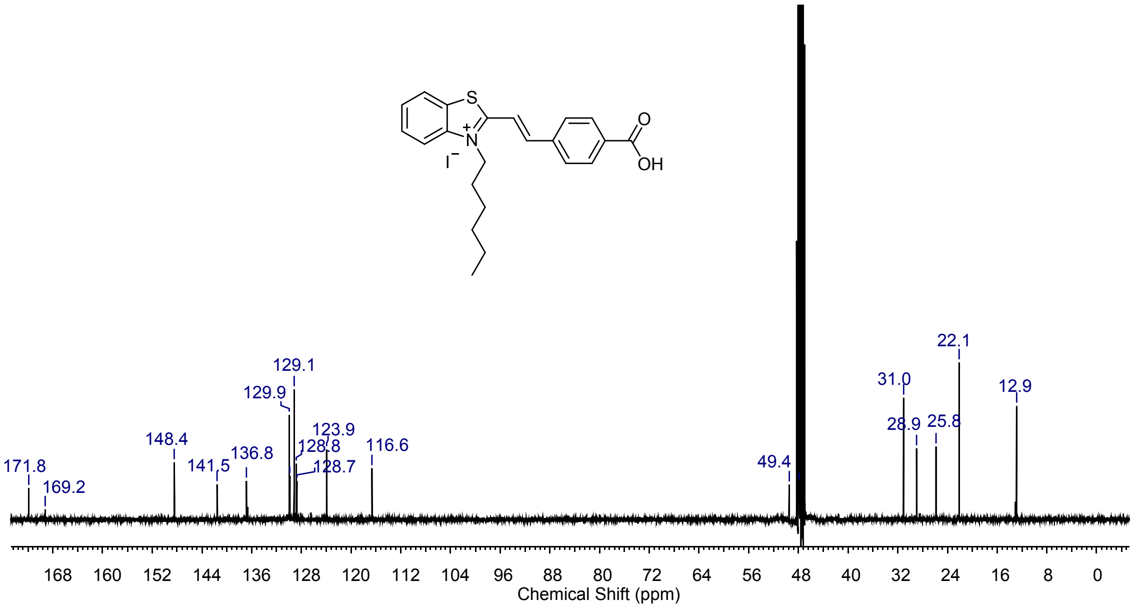

Figure S6. ^{13}C NMR spectrum (MeOD-d_4, 100 MHz) of merocyanine **6**.

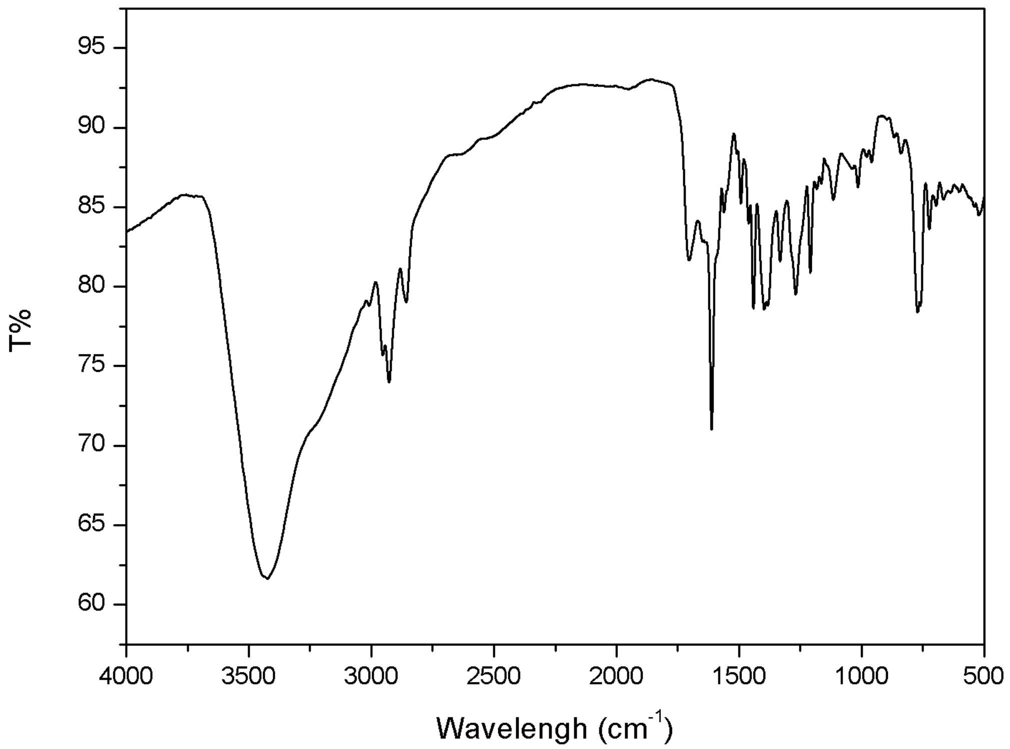

Figure S7. FTIR spectra of merocyanine **6**.

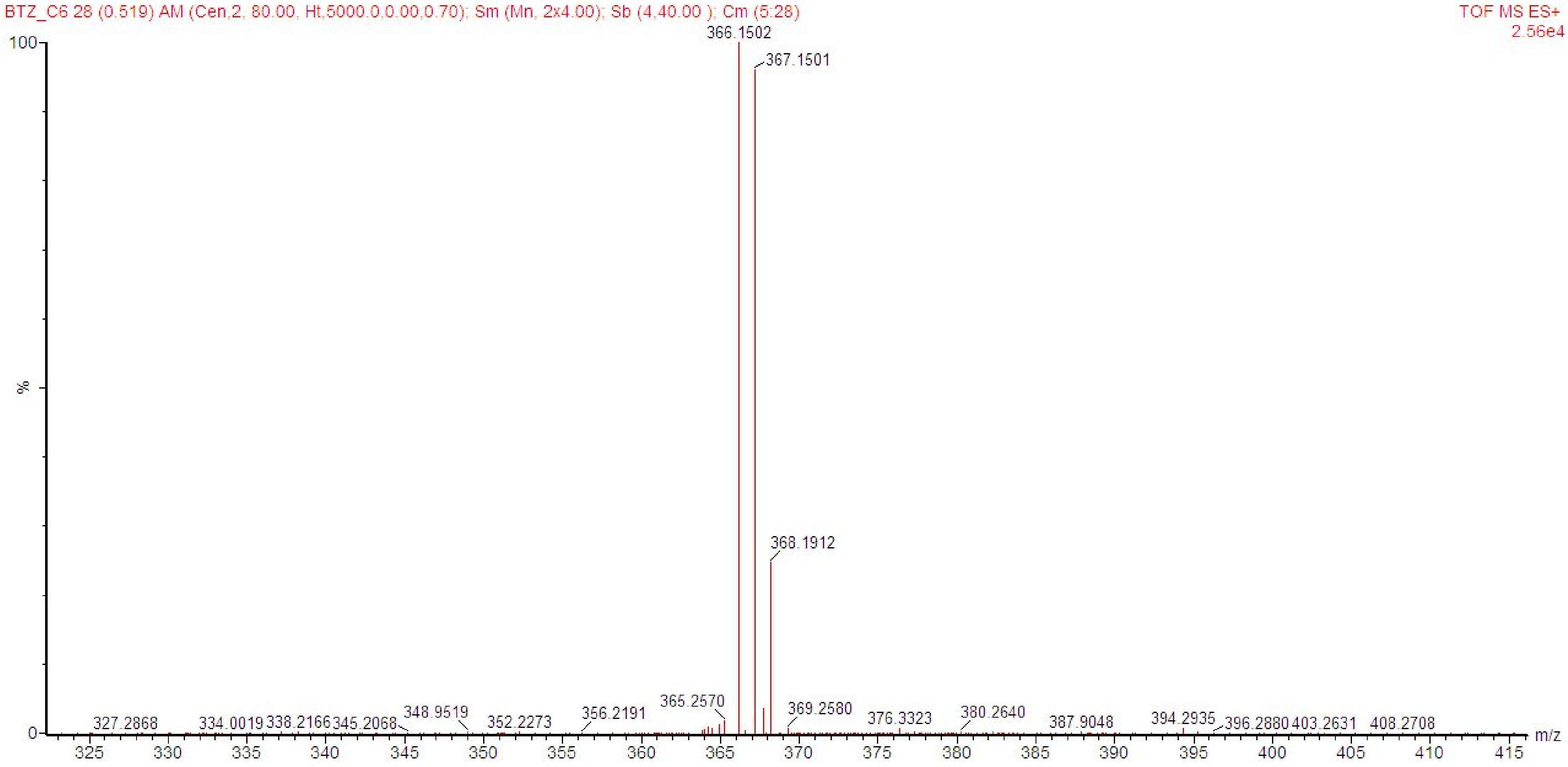

Figure S8. ESI-MS analysis of merocyanine **6**.

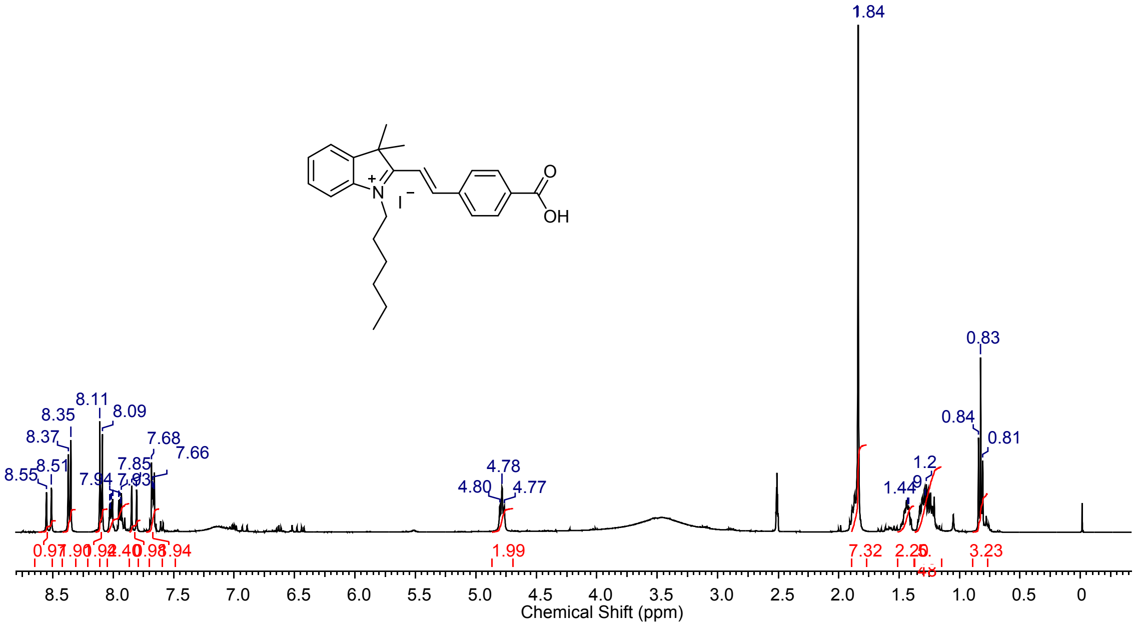

Figure S9. ^{1}H NMR spectrum (DMSO-d_6, 400 MHz) of merocyanine **7**.

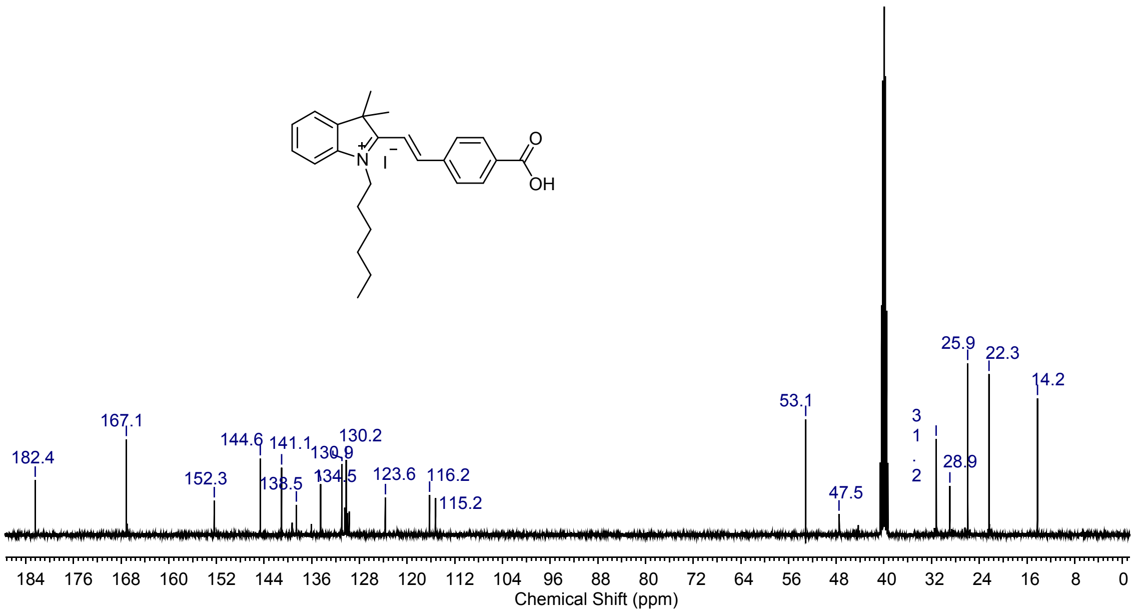

Figure S10. ^{13}C NMR spectrum (DMSO-d_6, 100 MHz) of merocyanine **7**.

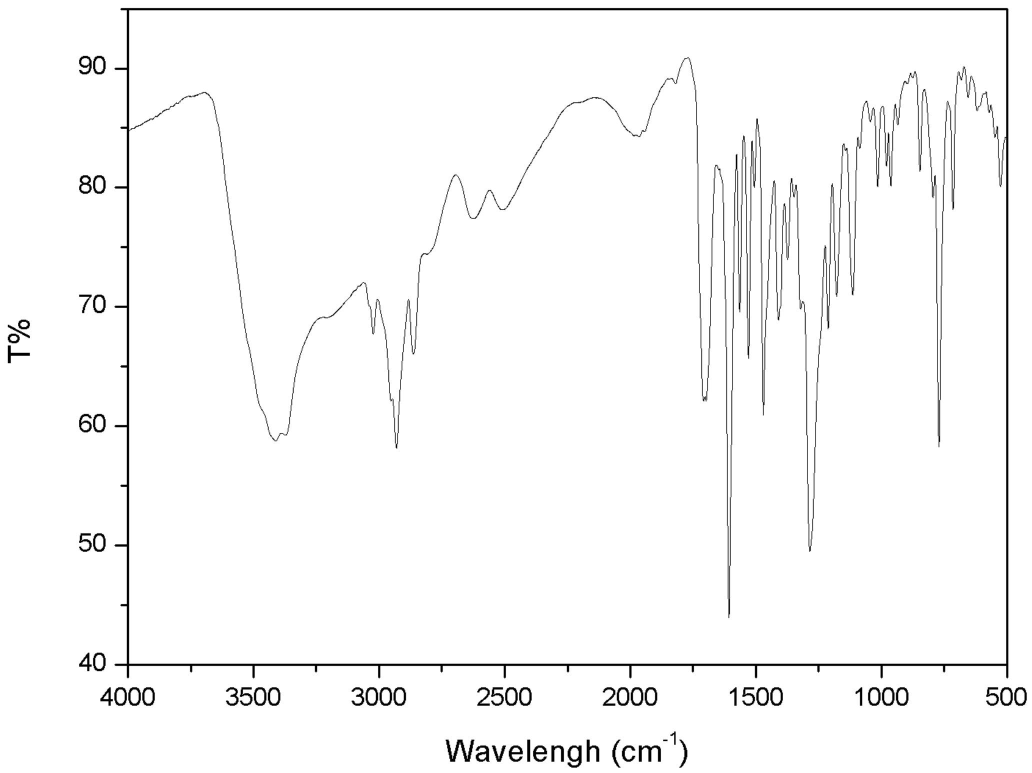

Figure S11. FTIR spectrum of merocyanine **7**.

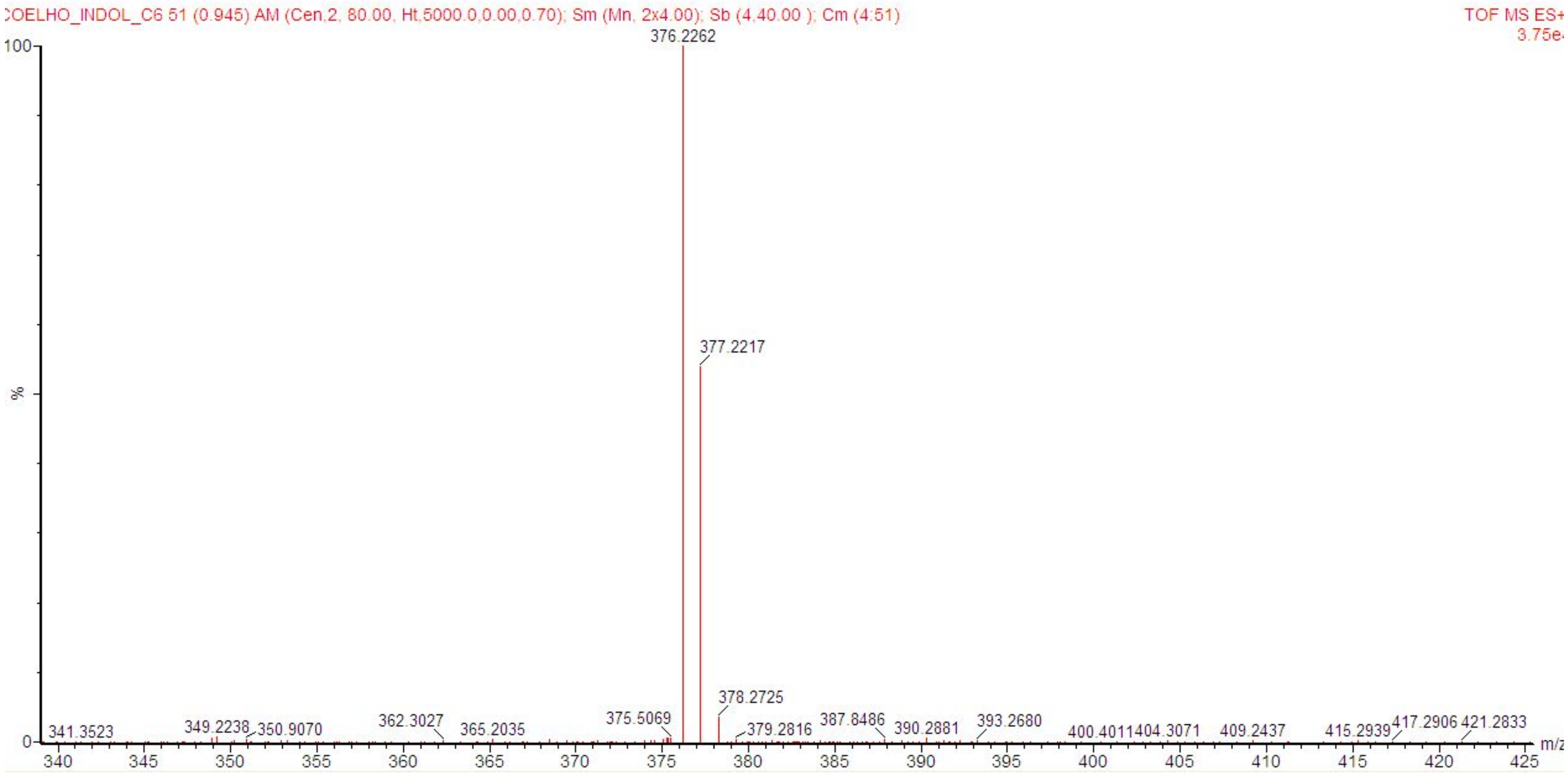

Figure S12. ESI-MS analysis of merocyanine **7**.

Additional photophysical data

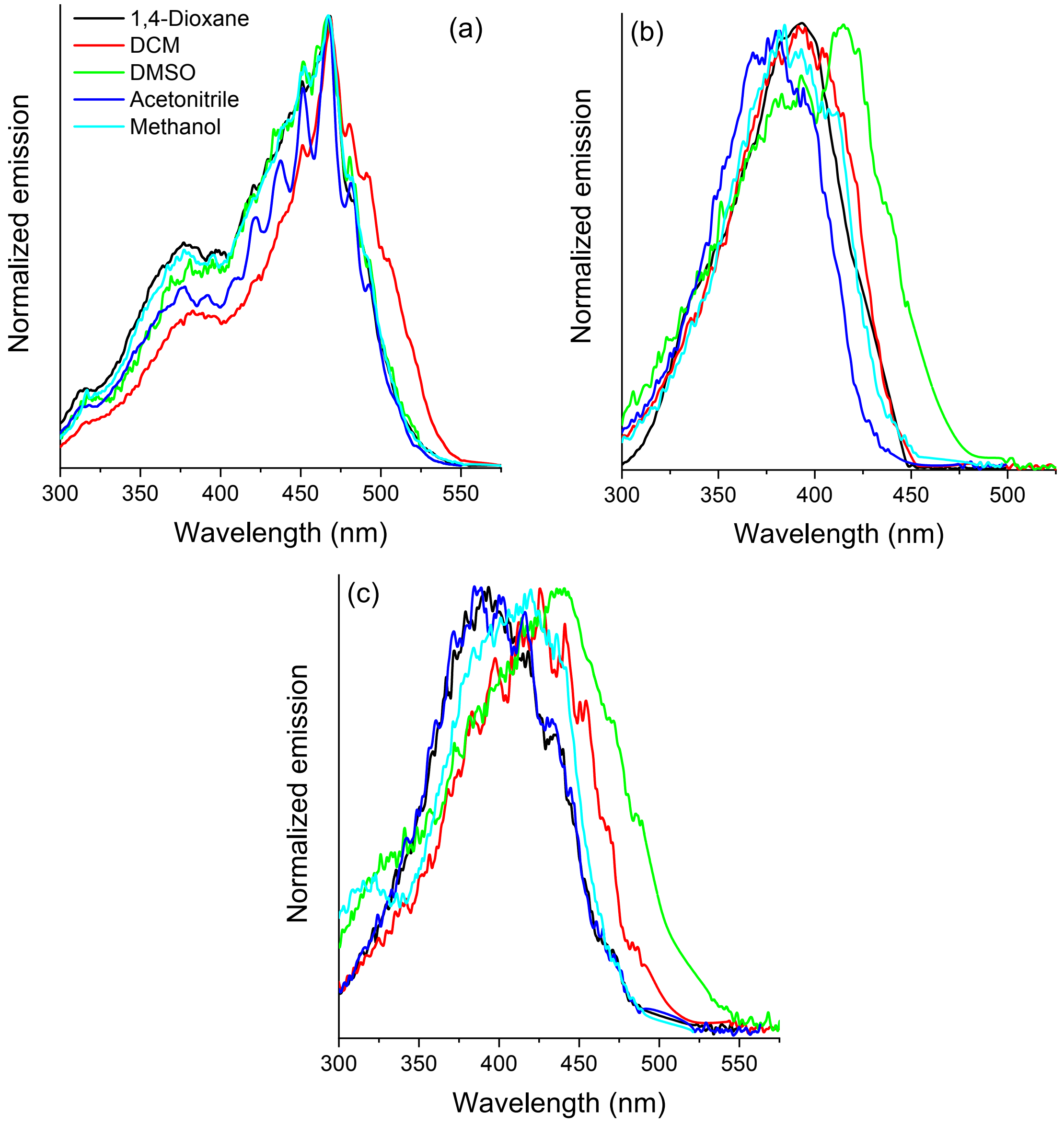

Figure S13. Excitation spectra of merocyanines (a) **3**, (b) **6**, and (c) **7** in different organic solvents (~10^{-5} M).

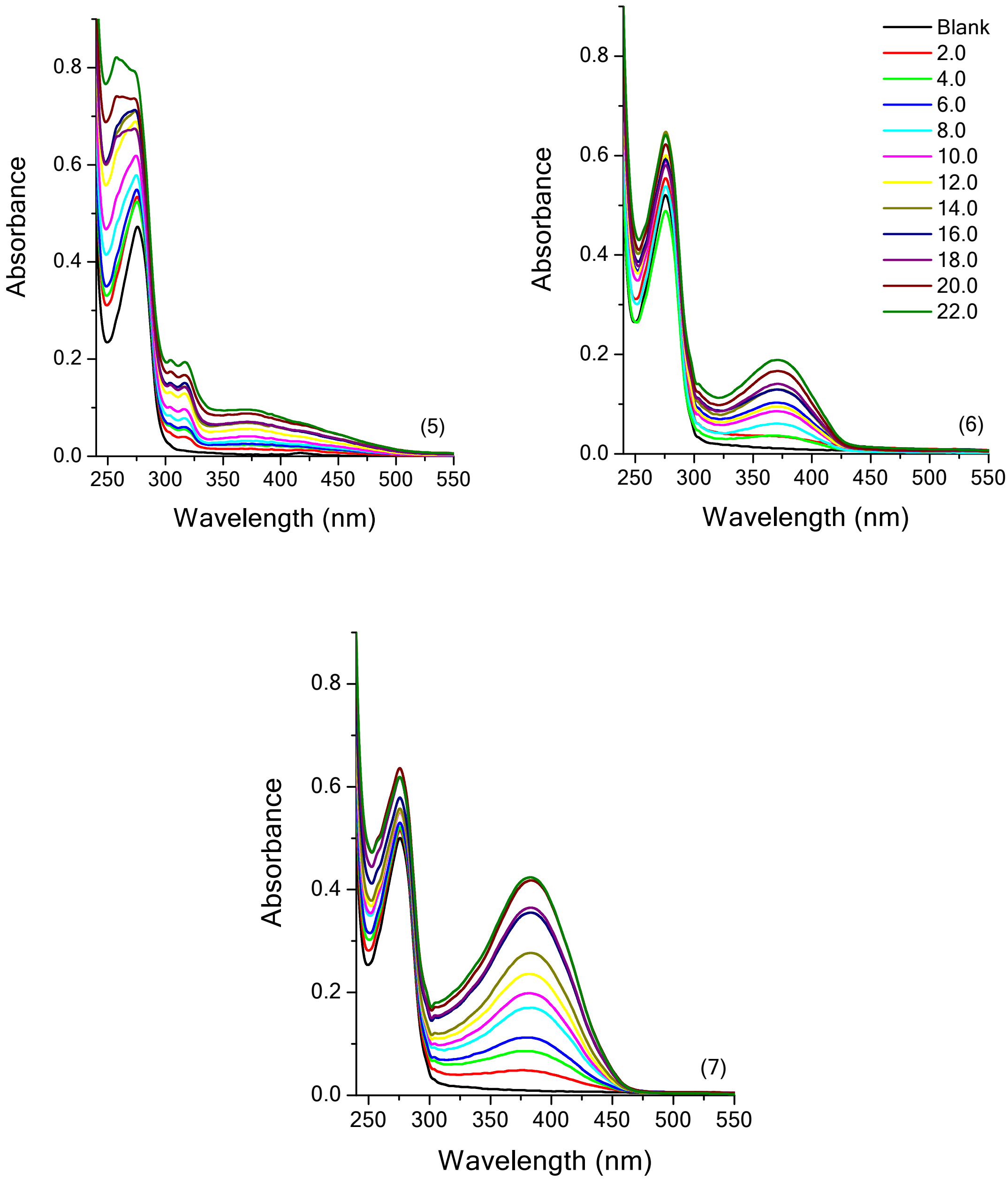

Figure S14. UV-Vis absorption and fluorescence emission spectra (λ_{exc}=279 nm) of BSA in PBS solution (11 μM) in the presence of different amounts of (a) **5**, (b) **6** and (c) **7** (μM). The blank sample is the pure BSA.

Theoretical Calculations

Merocyanine **3** - geometries

Acetonitrile

S_0

C	-1.542467000	-0.567361000	0.000162000
C	-2.924465000	0.058983000	0.000047000
C	-2.792333000	1.426197000	0.000063000
N	-1.409219000	1.755338000	0.000107000
C	-0.658510000	0.672425000	0.000127000
C	0.772632000	0.769629000	0.000097000
C	1.622527000	-0.283014000	0.000072000
C	3.080329000	-0.207851000	0.000029000
C	3.800708000	-1.411988000	-0.000010000
C	5.188951000	-1.411447000	-0.000059000
C	5.878230000	-0.198490000	-0.000069000
C	5.171542000	1.008370000	-0.000028000
C	3.787871000	1.006797000	0.000021000
H	3.257932000	1.958834000	0.000051000
H	5.729109000	1.944260000	-0.000036000
C	7.366865000	-0.138848000	-0.000124000
O	8.004513000	0.890016000	-0.000107000
O	7.937728000	-1.349104000	-0.000134000
H	8.902251000	-1.213738000	-0.000148000
H	5.740232000	-2.350216000	-0.000091000
H	3.260385000	-2.359913000	-0.000004000
H	1.226381000	-1.298010000	0.000071000
H	1.182595000	1.777844000	0.000076000
C	-0.906739000	3.128502000	0.000085000
H	-0.303424000	3.304462000	0.898626000
H	-0.303056000	3.304295000	-0.898239000
H	-1.752017000	3.818841000	-0.000162000
C	-3.879446000	2.321152000	0.000013000
C	-5.134227000	1.772418000	-0.000058000
C	-5.337736000	0.363868000	-0.000091000
C	-4.218932000	-0.527046000	-0.000042000
C	-4.469325000	-1.927458000	-0.000087000
C	-5.753148000	-2.412255000	-0.000168000
C	-6.857906000	-1.529730000	-0.000209000
C	-6.651297000	-0.174109000	-0.000173000
H	-7.497428000	0.515530000	-0.000207000
H	-7.872080000	-1.931413000	-0.000273000
H	-5.923138000	-3.489820000	-0.000202000
H	-3.638518000	-2.630323000	-0.000064000
H	-6.009045000	2.424021000	-0.000098000
H	-3.744556000	3.401002000	0.000031000
C	-1.305322000	-1.386915000	-1.285868000

H	-0.298934000	-1.821958000	-1.308798000
H	-2.028769000	-2.209072000	-1.342579000
H	-1.433453000	-0.759647000	-2.179080000
C	-1.305484000	-1.386755000	1.286328000
H	-1.433654000	-0.759355000	2.179442000
H	-2.028989000	-2.208856000	1.343093000
H	-0.299131000	-1.821867000	1.309413000

S_1

C	-1.551905000	-0.613805000	0.000033000
C	-2.915622000	0.048739000	0.000000000
C	-2.745118000	1.455527000	-0.000009000
N	-1.420543000	1.760906000	-0.000004000
C	-0.638937000	0.607582000	0.000026000
C	0.740652000	0.684544000	0.000035000
C	1.641003000	-0.391728000	0.000010000
C	3.055694000	-0.275172000	0.000012000
C	3.837920000	-1.465074000	-0.000020000
C	5.215653000	-1.417552000	-0.000021000
C	5.874863000	-0.178290000	0.000009000
C	5.124920000	1.008461000	0.000042000
C	3.748221000	0.969875000	0.000044000
H	3.193315000	1.907249000	0.000070000
H	5.655413000	1.960383000	0.000066000
C	7.353274000	-0.073745000	0.000009000
O	7.963593000	0.974299000	0.000030000
O	7.964202000	-1.267191000	-0.000013000
H	8.923239000	-1.098385000	-0.000011000
H	5.798169000	-2.337647000	-0.000046000
H	3.327190000	-2.429544000	-0.000044000
H	1.253808000	-1.409758000	-0.000022000
H	1.166917000	1.686981000	0.000049000
C	-0.870928000	3.103232000	-0.000028000
H	-0.253399000	3.258373000	0.895840000
H	-0.253231000	3.258263000	-0.895797000
H	-1.681870000	3.834747000	-0.000154000
C	-3.838543000	2.355988000	-0.000028000
C	-5.092855000	1.822803000	-0.000041000
C	-5.322601000	0.406912000	-0.000037000
C	-4.210311000	-0.501113000	-0.000016000
C	-4.485015000	-1.899573000	-0.000013000
C	-5.781113000	-2.365598000	-0.000030000
C	-6.861441000	-1.467272000	-0.000050000
C	-6.629401000	-0.100693000	-0.000054000
H	-7.468173000	0.597262000	-0.000070000
H	-7.884130000	-1.845943000	-0.000064000
H	-5.968434000	-3.439700000	-0.000029000
H	-3.666434000	-2.615104000	0.000000000

H	-5.961109000	2.483145000	-0.000055000
H	-3.690657000	3.433533000	-0.000030000
C	-1.342918000	-1.457047000	-1.279271000
H	-0.330465000	-1.877526000	-1.311353000
H	-2.052330000	-2.292973000	-1.314525000
H	-1.489268000	-0.843095000	-2.178718000
C	-1.342969000	-1.457005000	1.279374000
H	-1.489338000	-0.843019000	2.178794000
H	-2.052395000	-2.292919000	1.314636000
H	-0.330525000	-1.877502000	1.311504000

Dichloromethane

S_0

C	-1.543540000	-0.568764000	-0.000303000
C	-2.925155000	0.058787000	-0.000129000
C	-2.792002000	1.426014000	-0.000147000
N	-1.408252000	1.753712000	-0.000229000
C	-0.657839000	0.670045000	-0.000244000
C	0.772870000	0.766364000	-0.000177000
C	1.624250000	-0.285959000	-0.000091000
C	3.081226000	-0.210009000	-0.000005000
C	3.802560000	-1.413599000	0.000087000
C	5.190766000	-1.412184000	0.000173000
C	5.878612000	-0.198665000	0.000169000
C	5.171485000	1.007870000	0.000078000
C	3.788051000	1.005198000	-0.000008000
H	3.257340000	1.956859000	-0.000076000
H	5.729869000	1.943256000	0.000078000
C	7.367805000	-0.136456000	0.000260000
O	8.002247000	0.893434000	0.000252000
O	7.940048000	-1.346356000	0.000335000
H	8.904235000	-1.208988000	0.000384000
H	5.743625000	-2.350027000	0.000244000
H	3.262779000	-2.361841000	0.000091000
H	1.228081000	-1.301282000	-0.000063000
H	1.183749000	1.774345000	-0.000164000
C	-0.904337000	3.126036000	-0.000180000
H	-0.300402000	3.301902000	0.898118000
H	-0.300177000	3.301887000	-0.898327000
H	-1.748570000	3.817761000	-0.000301000
C	-3.878254000	2.321980000	-0.000044000
C	-5.133286000	1.773858000	0.000074000
C	-5.337957000	0.365610000	0.000112000
C	-4.219973000	-0.526351000	0.000020000
C	-4.471741000	-1.926316000	0.000089000
C	-5.755975000	-2.409729000	0.000225000
C	-6.859732000	-1.526247000	0.000302000
C	-6.651887000	-0.170961000	0.000249000

H	-7.497474000	0.519283000	0.000315000
H	-7.874256000	-1.926911000	0.000411000
H	-5.927231000	-3.487079000	0.000277000
H	-3.641881000	-2.630297000	0.000045000
H	-6.007713000	2.425994000	0.000158000
H	-3.743402000	3.401878000	-0.000044000
C	-1.307402000	-1.388157000	-1.286664000
H	-0.300367000	-1.821732000	-1.312142000
H	-2.030149000	-2.210984000	-1.342776000
H	-1.438480000	-0.761449000	-2.179896000
C	-1.307200000	-1.388389000	1.285869000
H	-1.438155000	-0.761853000	2.179239000
H	-2.029931000	-2.211236000	1.341924000
H	-0.300151000	-1.821952000	1.311109000

S_1

C	-1.550987000	-0.611964000	-0.000030000
C	-2.915694000	0.049640000	-0.000027000
C	-2.747146000	1.456464000	-0.000035000
N	-1.421145000	1.764354000	-0.000041000
C	-0.639387000	0.611135000	-0.000026000
C	0.740885000	0.688266000	-0.000001000
C	1.639986000	-0.387224000	0.000017000
C	3.056467000	-0.272876000	0.000048000
C	3.835908000	-1.463315000	0.000050000
C	5.214119000	-1.417927000	0.000076000
C	5.874277000	-0.179808000	0.000105000
C	5.126710000	1.007872000	0.000103000
C	3.749697000	0.970580000	0.000076000
H	3.195603000	1.908496000	0.000076000
H	5.659766000	1.958355000	0.000126000
C	7.353678000	-0.076126000	0.000143000
O	7.963371000	0.971257000	-0.000007000
O	7.963060000	-1.270605000	-0.000089000
H	8.922087000	-1.102062000	-0.000214000
H	5.796125000	-2.338307000	0.000078000
H	3.323632000	-2.427027000	0.000029000
H	1.252128000	-1.405110000	0.000004000
H	1.167399000	1.690737000	0.000009000
C	-0.872419000	3.106533000	-0.000047000
H	-0.254798000	3.263382000	0.895719000
H	-0.254650000	3.263304000	-0.895724000
H	-1.683545000	3.838040000	-0.000150000
C	-3.840582000	2.354684000	-0.000034000
C	-5.095271000	1.819926000	-0.000027000
C	-5.323452000	0.404401000	-0.000021000
C	-4.209807000	-0.501797000	-0.000021000
C	-4.482981000	-1.900424000	-0.000015000

C	-5.778783000	-2.368599000	-0.000009000
C	-6.859858000	-1.472251000	-0.000008000
C	-6.629311000	-0.104832000	-0.000014000
H	-7.469376000	0.591576000	-0.000014000
H	-7.882118000	-1.851986000	-0.000003000
H	-5.964460000	-3.442975000	-0.000004000
H	-3.663618000	-2.615000000	-0.000015000
H	-5.964004000	2.479735000	-0.000025000
H	-3.694677000	3.432535000	-0.000035000
C	-1.341118000	-1.454959000	-1.279432000
H	-0.327231000	-1.871745000	-1.314048000
H	-2.047587000	-2.293409000	-1.314152000
H	-1.491108000	-0.841819000	-2.178847000
C	-1.341113000	-1.454968000	1.279364000
H	-1.491101000	-0.841836000	2.178784000
H	-2.047579000	-2.293420000	1.314079000
H	-0.327224000	-1.871751000	1.313973000

1,4-dioxane

S_0

C	-1.543925000	-0.568203000	0.000111000
C	-2.925999000	0.059345000	0.000019000
C	-2.793434000	1.426865000	0.000041000
N	-1.408698000	1.754241000	0.000094000
C	-0.656045000	0.670038000	0.000089000
C	0.773047000	0.766170000	0.000052000
C	1.628387000	-0.285769000	0.000065000
C	3.082661000	-0.211678000	0.000023000
C	3.803774000	-1.415911000	0.000035000
C	5.191906000	-1.415045000	-0.000001000
C	5.878479000	-0.201389000	-0.000052000
C	5.173104000	1.006075000	-0.000065000
C	3.790383000	1.003590000	-0.000027000
H	3.259699000	1.955549000	-0.000035000
H	5.736177000	1.938769000	-0.000104000
C	7.369542000	-0.134602000	-0.000094000
O	7.995938000	0.897242000	-0.000122000
O	7.942932000	-1.344748000	-0.000068000
H	8.906491000	-1.203874000	-0.000091000
H	5.747003000	-2.351718000	0.000007000
H	3.263985000	-2.364542000	0.000069000
H	1.229646000	-1.300619000	0.000108000
H	1.185010000	1.774095000	-0.000003000
C	-0.905661000	3.126040000	0.000148000
H	-0.301165000	3.304562000	0.898111000
H	-0.300907000	3.304541000	-0.897642000
H	-1.749860000	3.818232000	0.000010000
C	-3.879858000	2.322505000	-0.000003000

C	-5.134136000	1.772613000	-0.000070000
C	-5.338327000	0.364683000	-0.000092000
C	-4.220077000	-0.527032000	-0.000046000
C	-4.471616000	-1.926571000	-0.000063000
C	-5.755715000	-2.409703000	-0.000121000
C	-6.859249000	-1.526636000	-0.000168000
C	-6.651813000	-0.171656000	-0.000155000
H	-7.497892000	0.517799000	-0.000191000
H	-7.873585000	-1.927351000	-0.000215000
H	-5.927605000	-3.486878000	-0.000131000
H	-3.642168000	-2.631098000	-0.000029000
H	-6.009293000	2.423756000	-0.000108000
H	-3.748109000	3.402907000	0.000015000
C	-1.308859000	-1.386715000	-1.287090000
H	-0.301521000	-1.819912000	-1.316884000
H	-2.032045000	-2.209114000	-1.345161000
H	-1.442571000	-0.759556000	-2.179773000
C	-1.309010000	-1.386551000	1.287451000
H	-1.442774000	-0.759264000	2.180035000
H	-2.032244000	-2.208905000	1.345569000
H	-0.301700000	-1.819800000	1.317401000

S_1

C	-1.548294000	-0.606010000	0.000027000
C	-2.915857000	0.053291000	-0.000018000
C	-2.753333000	1.460000000	-0.000010000
N	-1.422618000	1.775920000	0.000017000
C	-0.640623000	0.622721000	0.000019000
C	0.741879000	0.699007000	0.000027000
C	1.637907000	-0.374294000	0.000067000
C	3.059063000	-0.266787000	0.000061000
C	3.830566000	-1.458944000	0.000096000
C	5.210213000	-1.419733000	0.000085000
C	5.873041000	-0.184900000	0.000043000
C	5.132272000	1.005558000	0.000011000
C	3.754486000	0.972236000	0.000019000
H	3.203161000	1.912125000	-0.000006000
H	5.672575000	1.952033000	-0.000018000
C	7.355353000	-0.082888000	0.000036000
O	7.962146000	0.963001000	-0.000117000
O	7.960805000	-1.280179000	-0.000111000
H	8.919626000	-1.111564000	-0.000231000
H	5.790929000	-2.340917000	0.000109000
H	3.314107000	-2.420816000	0.000128000
H	1.247575000	-1.391452000	0.000110000
H	1.169734000	1.701264000	0.000003000
C	-0.877130000	3.117610000	0.000068000
H	-0.259363000	3.279859000	0.895619000

H	-0.259272000	3.279891000	-0.895413000
H	-1.689246000	3.848666000	0.000036000
C	-3.846549000	2.350342000	-0.000025000
C	-5.103290000	1.810407000	-0.000060000
C	-5.326332000	0.396715000	-0.000069000
C	-4.208667000	-0.503299000	-0.000040000
C	-4.477076000	-1.902009000	-0.000024000
C	-5.772686000	-2.377192000	-0.000052000
C	-6.856288000	-1.487798000	-0.000093000
C	-6.629992000	-0.117615000	-0.000097000
H	-7.473734000	0.574459000	-0.000119000
H	-7.877164000	-1.871083000	-0.000115000
H	-5.952814000	-3.452513000	-0.000041000
H	-3.655295000	-2.613798000	0.000018000
H	-5.973443000	2.468627000	-0.000077000
H	-3.707132000	3.429186000	-0.000011000
C	-1.335978000	-1.449493000	-1.278997000
H	-0.320571000	-1.862515000	-1.316016000
H	-2.038594000	-2.291178000	-1.315259000
H	-1.488338000	-0.838114000	-2.179176000
C	-1.336055000	-1.449387000	1.279134000
H	-1.488464000	-0.837927000	2.179251000
H	-2.038686000	-2.291059000	1.315423000
H	-0.320652000	-1.862413000	1.316241000

Merocyanine **6** - geometries

Acetonitrile

S_0

C	2.360849000	2.504615000	-0.002659000
N	2.666476000	1.069675000	-0.001597000
C	1.717574000	0.134367000	-0.001074000
S	2.364012000	-1.465535000	0.000251000
C	3.991831000	-0.832259000	0.000324000
C	3.974365000	0.565269000	-0.000238000
C	5.169861000	1.288211000	0.000668000
C	6.358518000	0.576274000	0.001681000
C	6.371201000	-0.826503000	0.001885000
C	5.188213000	-1.548290000	0.001258000
H	5.188121000	-2.637742000	0.001629000
H	7.323479000	-1.357259000	0.002702000
H	7.302045000	1.122184000	0.002425000
H	5.182326000	2.376282000	0.000830000
C	0.309480000	0.437119000	-0.000692000
C	-0.645836000	-0.515498000	-0.001899000
C	-2.089095000	-0.297405000	-0.001146000
C	-2.926382000	-1.422906000	-0.002017000
C	-4.307619000	-1.282308000	-0.001257000

C	-4.871617000	-0.006346000	0.000364000
C	-4.046938000	1.123213000	0.001195000
C	-2.670411000	0.982240000	0.000448000
H	-2.046630000	1.875811000	0.001084000
H	-4.507585000	2.110475000	0.002432000
C	-6.346437000	0.202229000	0.001259000
O	-6.878326000	1.289623000	0.002720000
O	-7.035895000	-0.944873000	0.000271000
H	-7.981915000	-0.713406000	0.000964000
H	-4.950587000	-2.160857000	-0.001907000
H	-2.484661000	-2.420666000	-0.003262000
H	-0.346249000	-1.567751000	-0.003520000
H	0.042244000	1.491696000	0.001234000
H	3.296235000	3.066236000	-0.016590000
H	1.780308000	2.759725000	-0.896910000
H	1.801767000	2.765982000	0.903535000

S_1

C	2.369480000	2.531176000	-0.002123000
N	2.681411000	1.108715000	-0.002297000
C	1.679666000	0.158901000	-0.002555000
S	2.346351000	-1.470720000	-0.002574000
C	3.958567000	-0.830514000	-0.000369000
C	3.948584000	0.589458000	-0.000279000
C	5.165326000	1.292874000	0.001741000
C	6.340898000	0.567974000	0.003238000
C	6.342656000	-0.842471000	0.002920000
C	5.153345000	-1.550559000	0.001156000
H	5.143108000	-2.640065000	0.001011000
H	7.291288000	-1.379580000	0.004168000
H	7.291676000	1.101766000	0.004817000
H	5.189612000	2.380638000	0.002292000
C	0.324890000	0.426146000	-0.001885000
C	-0.670972000	-0.583321000	-0.002033000
C	-2.055320000	-0.350977000	-0.001014000
C	-2.945810000	-1.474233000	-0.001086000
C	-4.308349000	-1.301312000	-0.000068000
C	-4.852155000	-0.002249000	0.001014000
C	-4.001660000	1.117721000	0.001058000
C	-2.637156000	0.959844000	0.000098000
H	-1.999118000	1.842354000	0.000139000
H	-4.449542000	2.110908000	0.001877000
C	-6.316902000	0.233691000	0.002126000
O	-6.826124000	1.333928000	0.003040000
O	-7.028214000	-0.900642000	0.002151000
H	-7.970034000	-0.652358000	0.002950000
H	-4.974517000	-2.162454000	-0.000094000
H	-2.520785000	-2.479085000	-0.001924000

H	-0.350578000	-1.627770000	-0.002988000
H	0.021720000	1.471783000	-0.000879000
H	3.296824000	3.107518000	-0.012061000
H	1.783729000	2.786891000	-0.895649000
H	1.799303000	2.790796000	0.900433000

Dichloromethane

S_0

C	2.362111000	2.505145000	-0.002456000
N	2.667193000	1.070352000	-0.000903000
C	1.717025000	0.135663000	0.000268000
S	2.363259000	-1.464524000	0.002073000
C	3.991895000	-0.832320000	0.000631000
C	3.975324000	0.565209000	-0.000417000
C	5.171376000	1.287114000	-0.000924000
C	6.359470000	0.574114000	-0.000595000
C	6.371060000	-0.828489000	0.000251000
C	5.187501000	-1.549379000	0.000886000
H	5.186967000	-2.638993000	0.001697000
H	7.322926000	-1.359904000	0.000519000
H	7.303520000	1.119060000	-0.000952000
H	5.185221000	2.375317000	-0.001472000
C	0.309723000	0.438722000	0.000853000
C	-0.646945000	-0.513507000	-0.000918000
C	-2.089462000	-0.296405000	-0.000464000
C	-2.926070000	-1.422554000	-0.002441000
C	-4.307335000	-1.282727000	-0.002197000
C	-4.871549000	-0.007135000	0.000046000
C	-4.048057000	1.123196000	0.002026000
C	-2.671661000	0.983044000	0.001760000
H	-2.048672000	1.877308000	0.003271000
H	-4.510844000	2.109463000	0.003745000
C	-6.347045000	0.201698000	0.000402000
O	-6.877538000	1.288861000	0.002452000
O	-7.035813000	-0.946077000	-0.001851000
H	-7.981601000	-0.714004000	-0.001434000
H	-4.950511000	-2.161123000	-0.003742000
H	-2.484218000	-2.420356000	-0.004193000
H	-0.346856000	-1.565792000	-0.002969000
H	0.041891000	1.493144000	0.003051000
H	3.297662000	3.066717000	-0.012965000
H	1.784461000	2.761423000	-0.898486000
H	1.800185000	2.767029000	0.902019000

S_1

C	2.370507000	2.532018000	-0.001122000
N	2.682201000	1.109825000	-0.001253000
C	1.680033000	0.160996000	-0.001177000

S	2.345087000	-1.469257000	-0.001004000
C	3.958453000	-0.830410000	-0.000059000
C	3.950005000	0.589351000	-0.000270000
C	5.167100000	1.291435000	0.000421000
C	6.342127000	0.565269000	0.001230000
C	6.342461000	-0.845048000	0.001423000
C	5.152403000	-1.551787000	0.000774000
H	5.141286000	-2.641437000	0.000926000
H	7.290538000	-1.383096000	0.002102000
H	7.293469000	1.098037000	0.001774000
H	5.192801000	2.379334000	0.000332000
C	0.324177000	0.428171000	-0.000692000
C	-0.669868000	-0.581459000	-0.000686000
C	-2.056175000	-0.350302000	-0.000281000
C	-2.945282000	-1.473803000	-0.000331000
C	-4.308103000	-1.301767000	0.000010000
C	-4.852179000	-0.003052000	0.000400000
C	-4.002911000	1.117390000	0.000445000
C	-2.638207000	0.959794000	0.000126000
H	-2.000549000	1.842784000	0.000158000
H	-4.452542000	2.109838000	0.000738000
C	-6.317878000	0.233129000	0.000757000
O	-6.825447000	1.333154000	0.001272000
O	-7.028493000	-0.901694000	0.000899000
H	-7.970252000	-0.653303000	0.001300000
H	-4.974417000	-2.162821000	-0.000019000
H	-2.519866000	-2.478603000	-0.000631000
H	-0.348155000	-1.625587000	-0.001083000
H	0.019747000	1.473494000	-0.000150000
H	3.297748000	3.108698000	-0.006376000
H	1.788336000	2.789377000	-0.896737000
H	1.796611000	2.791382000	0.899309000

1,4-dioxane

S_0

C	2.370077000	2.507780000	-0.000565000
N	2.670896000	1.073176000	-0.000197000
C	1.715263000	0.141642000	0.000247000
S	2.359152000	-1.460587000	0.000950000
C	3.991095000	-0.833399000	0.000424000
C	3.978981000	0.564058000	-0.000183000
C	5.177705000	1.281409000	-0.000724000
C	6.363332000	0.564095000	-0.000603000
C	6.369973000	-0.838105000	0.000055000
C	5.183779000	-1.554696000	0.000571000
H	5.180771000	-2.644690000	0.001025000
H	7.320270000	-1.372434000	0.000141000
H	7.309841000	1.104970000	-0.001007000

H	5.197170000	2.370048000	-0.001198000
C	0.310972000	0.447549000	0.000238000
C	-0.650796000	-0.503060000	-0.000375000
C	-2.090576000	-0.289758000	-0.000368000
C	-2.923763000	-1.419195000	-0.001108000
C	-4.305216000	-1.283764000	-0.000986000
C	-4.871824000	-0.009886000	-0.000088000
C	-4.053451000	1.124177000	0.000617000
C	-2.677280000	0.988568000	0.000458000
H	-2.057800000	1.885756000	0.001002000
H	-4.524383000	2.106722000	0.001305000
C	-6.349876000	0.197635000	0.000218000
O	-6.877050000	1.283514000	0.001250000
O	-7.034606000	-0.953307000	-0.000756000
H	-7.980255000	-0.721516000	-0.000421000
H	-4.947920000	-2.162551000	-0.001553000
H	-2.479552000	-2.416269000	-0.001784000
H	-0.349384000	-1.555534000	-0.000856000
H	0.042029000	1.501772000	0.000564000
H	3.307233000	3.067446000	-0.001165000
H	1.800668000	2.770427000	-0.900655000
H	1.801485000	2.771067000	0.899869000

S_1

C	2.376543000	2.535380000	-0.000759000
N	2.684819000	1.113490000	-0.000509000
C	1.681475000	0.168154000	-0.000401000
S	2.340309000	-1.464343000	0.000062000
C	3.957762000	-0.830477000	0.000164000
C	3.954658000	0.588436000	-0.000227000
C	5.172612000	1.286208000	-0.000264000
C	6.346042000	0.556023000	0.000125000
C	6.341755000	-0.853860000	0.000550000
C	5.149273000	-1.556144000	0.000564000
H	5.135754000	-2.646139000	0.000851000
H	7.288417000	-1.394681000	0.000840000
H	7.299398000	1.085526000	0.000110000
H	5.203042000	2.374515000	-0.000576000
C	0.321729000	0.434618000	-0.000529000
C	-0.666461000	-0.575067000	-0.000417000
C	-2.059278000	-0.347866000	-0.000299000
C	-2.943530000	-1.472203000	-0.000244000
C	-4.307463000	-1.303084000	-0.000015000
C	-4.852748000	-0.005452000	0.000185000
C	-4.007552000	1.116831000	0.000082000
C	-2.642005000	0.960070000	-0.000175000
H	-2.005594000	1.844490000	-0.000241000
H	-4.462973000	2.106844000	0.000225000

C	-6.321228000	0.231255000	0.000547000
O	-6.823612000	1.330658000	0.000814000
O	-7.028953000	-0.905617000	0.000576000
H	-7.970568000	-0.657209000	0.000835000
H	-4.974136000	-2.164002000	0.000035000
H	-2.516846000	-2.476742000	-0.000375000
H	-0.342206000	-1.618673000	-0.000395000
H	0.014167000	1.479256000	-0.000699000
H	3.305200000	3.110355000	-0.000887000
H	1.799325000	2.796962000	-0.899036000
H	1.799361000	2.797298000	0.897449000

Merocyanine **7** - geometries

Acetonitrile

S_0

C	-2.609634000	-0.160548000	0.000000000
C	-3.794268000	0.779470000	0.000000000
C	-3.340542000	2.092626000	0.000000000
N	-1.917338000	2.069588000	0.000000000
C	-1.454108000	0.834843000	0.000000000
C	-0.043488000	0.579104000	0.000000000
C	0.509109000	-0.656008000	0.000000000
C	1.936099000	-0.960283000	0.000000000
C	2.319644000	-2.310030000	0.000000000
C	3.660577000	-2.669080000	0.000000000
C	4.640157000	-1.675712000	0.000000000
C	4.270552000	-0.326886000	0.000000000
C	2.933696000	0.030115000	0.000000000
H	2.668118000	1.086885000	0.000000000
H	5.051933000	0.432234000	0.000000000
C	6.093594000	-2.003878000	0.000000000
O	6.975923000	-1.175371000	0.000000000
O	6.330933000	-3.320727000	0.000000000
H	7.297490000	-3.440654000	0.000000000
H	3.950116000	-3.718536000	0.000000000
H	1.551939000	-3.085330000	0.000000000
H	-0.137056000	-1.533411000	0.000000000
H	0.605530000	1.452843000	0.000000000
C	-1.096528000	3.279006000	0.000000000
H	-0.468430000	3.303780000	-0.898646000
H	-1.751446000	4.152414000	0.000000000
C	-4.192322000	3.187341000	0.000000000
C	-5.560336000	2.917880000	0.000000000
C	-6.037093000	1.605667000	0.000000000
C	-5.156456000	0.522420000	0.000000000
H	-5.538064000	-0.499427000	0.000000000
H	-7.112521000	1.424511000	0.000000000

H	-6.265413000	3.749595000	0.000000000
H	-3.830680000	4.214445000	0.000000000
C	-2.609634000	-1.023912000	-1.276666000
H	-1.757371000	-1.714187000	-1.305258000
H	-3.530302000	-1.622840000	-1.300139000
H	-2.583766000	-0.398381000	-2.179743000

S_1

C	-2.622929000	0.303457000	0.000000000
C	-3.570961000	1.477728000	0.000000000
C	-2.830698000	2.686859000	0.000000000
N	-1.497585000	2.398747000	0.000000000
C	-1.278846000	1.022498000	0.000000000
C	0.002684000	0.507797000	0.000000000
C	0.348513000	-0.861382000	0.000000000
C	1.667926000	-1.361477000	0.000000000
C	1.864435000	-2.777594000	0.000000000
C	3.126958000	-3.323522000	0.000000000
C	4.254753000	-2.483506000	0.000000000
C	4.089549000	-1.087925000	0.000000000
C	2.832361000	-0.530953000	0.000000000
H	2.732763000	0.553529000	0.000000000
H	4.978797000	-0.458288000	0.000000000
C	5.635878000	-3.024574000	0.000000000
O	6.634761000	-2.337379000	0.000000000
O	5.672966000	-4.363521000	0.000000000
H	6.610696000	-4.626566000	0.000000000
H	3.261087000	-4.404053000	0.000000000
H	0.988180000	-3.427696000	0.000000000
H	-0.445695000	-1.606818000	0.000000000
H	0.819560000	1.228064000	0.000000000
C	-0.434876000	3.384637000	0.000000000
H	0.191411000	3.265364000	-0.895590000
H	-0.865300000	4.388817000	0.000000000
C	-3.466709000	3.944382000	0.000000000
C	-4.847406000	3.953942000	0.000000000
C	-5.589500000	2.755754000	0.000000000
C	-4.951303000	1.513202000	0.000000000
H	-5.537377000	0.593388000	0.000000000
H	-6.679015000	2.803092000	0.000000000
H	-5.375273000	4.907951000	0.000000000
H	-2.902476000	4.875077000	0.000000000
C	-2.830698000	-0.546899000	-1.270977000
H	-2.142434000	-1.401212000	-1.301023000
H	-3.856959000	-0.940334000	-1.282666000
H	-2.679662000	0.054348000	-2.178271000

Dichloromethane

S_0

C	-2.610689000	-0.162373000	0.000000000
C	-3.795453000	0.777907000	0.000000000
C	-3.341851000	2.091141000	0.000000000
N	-1.918337000	2.067874000	0.000000000
C	-1.454557000	0.832779000	0.000000000
C	-0.044406000	0.577643000	0.000000000
C	0.510904000	-0.657040000	0.000000000
C	1.937288000	-0.959581000	0.000000000
C	2.321505000	-2.309121000	0.000000000
C	3.662433000	-2.667848000	0.000000000
C	4.640991000	-1.673746000	0.000000000
C	4.271253000	-0.325003000	0.000000000
C	2.934465000	0.031366000	0.000000000
H	2.668312000	1.088004000	0.000000000
H	5.053642000	0.433043000	0.000000000
C	6.095609000	-1.999907000	0.000000000
O	6.975306000	-1.169765000	0.000000000
O	6.333938000	-3.316798000	0.000000000
H	7.300652000	-3.435011000	0.000000000
H	3.953409000	-3.716901000	0.000000000
H	1.553617000	-3.084097000	0.000000000
H	-0.134018000	-1.535731000	0.000000000
H	0.604348000	1.451797000	0.000000000
C	-1.097144000	3.276776000	0.000000000
H	-0.468517000	3.301674000	-0.898384000
H	-1.751018000	4.150988000	0.000000000
C	-4.193683000	3.185798000	0.000000000
C	-5.561598000	2.916487000	0.000000000
C	-6.038259000	1.604411000	0.000000000
C	-5.157725000	0.521241000	0.000000000
H	-5.540121000	-0.500277000	0.000000000
H	-7.113649000	1.423309000	0.000000000
H	-6.266678000	3.748156000	0.000000000
H	-3.832963000	4.213296000	0.000000000
C	-2.610689000	-1.025555000	-1.276892000
H	-1.756585000	-1.713544000	-1.308188000
H	-3.529612000	-1.627191000	-1.299401000
H	-2.588958000	-0.399990000	-2.180049000

S_1

C	-2.621775000	0.305758000	0.000000000
C	-3.569716000	1.480166000	0.000000000
C	-2.829925000	2.689611000	0.000000000
N	-1.495739000	2.401612000	0.000000000
C	-1.277536000	1.024870000	0.000000000
C	0.003883000	0.508507000	0.000000000

C	0.348086000	-0.860033000	0.000000000
C	1.668073000	-1.362714000	0.000000000
C	1.862589000	-2.778378000	0.000000000
C	3.124631000	-3.325944000	0.000000000
C	4.252808000	-2.487014000	0.000000000
C	4.089742000	-1.091587000	0.000000000
C	2.832782000	-0.533558000	0.000000000
H	2.734526000	0.551185000	0.000000000
H	4.980521000	-0.464052000	0.000000000
C	5.634475000	-3.029205000	0.000000000
O	6.632412000	-2.342190000	0.000000000
O	5.669415000	-4.368330000	0.000000000
H	6.606857000	-4.632154000	0.000000000
H	3.258383000	-4.406514000	0.000000000
H	0.985902000	-3.427997000	0.000000000
H	-0.447248000	-1.604433000	0.000000000
H	0.821727000	1.227669000	0.000000000
C	-0.432852000	3.386835000	0.000000000
H	0.193990000	3.268190000	-0.895446000
H	-0.862308000	4.391593000	0.000000000
C	-3.466381000	3.946525000	0.000000000
C	-4.847075000	3.955575000	0.000000000
C	-5.588962000	2.756937000	0.000000000
C	-4.950177000	1.514935000	0.000000000
H	-5.535739000	0.594611000	0.000000000
H	-6.678499000	2.803925000	0.000000000
H	-5.375489000	4.909276000	0.000000000
H	-2.902858000	4.877687000	0.000000000
C	-2.829925000	-0.544401000	-1.271187000
H	-2.142195000	-1.399121000	-1.302190000
H	-3.856432000	-0.937101000	-1.283526000
H	-2.678943000	0.056610000	-2.178644000

1,4-dioxane

S_0

C	-2.613491000	-0.160946000	0.000000000
C	-3.796821000	0.782495000	0.000000000
C	-3.340698000	2.094986000	0.000000000
N	-1.916364000	2.068309000	0.000000000
C	-1.453377000	0.830960000	0.000000000
C	-0.045780000	0.573706000	0.000000000
C	0.513950000	-0.661675000	0.000000000
C	1.937163000	-0.964152000	0.000000000
C	2.320427000	-2.314483000	0.000000000
C	3.660788000	-2.674634000	0.000000000
C	4.638641000	-1.680470000	0.000000000
C	4.271528000	-0.331038000	0.000000000
C	2.935828000	0.026189000	0.000000000

H	2.670606000	1.083174000	0.000000000
H	5.058635000	0.422153000	0.000000000
C	6.096360000	-2.003052000	0.000000000
O	6.968772000	-1.168944000	0.000000000
O	6.335486000	-3.320508000	0.000000000
H	7.302485000	-3.435207000	0.000000000
H	3.953938000	-3.723149000	0.000000000
H	1.551179000	-3.088289000	0.000000000
H	-0.131124000	-1.540976000	0.000000000
H	0.604812000	1.446898000	0.000000000
C	-1.091725000	3.273998000	0.000000000
H	-0.461345000	3.298417000	-0.897673000
H	-1.741229000	4.151842000	0.000000000
C	-4.190415000	3.191305000	0.000000000
C	-5.558748000	2.925416000	0.000000000
C	-6.037957000	1.614690000	0.000000000
C	-5.159921000	0.529836000	0.000000000
H	-5.546729000	-0.490027000	0.000000000
H	-7.113621000	1.435849000	0.000000000
H	-6.262142000	3.758402000	0.000000000
H	-3.829505000	4.218938000	0.000000000
C	-2.613491000	-1.021899000	-1.278811000
H	-1.753238000	-1.702042000	-1.319316000
H	-3.526991000	-1.631796000	-1.301877000
H	-2.603144000	-0.393656000	-2.180328000

S_1

C	-2.617898000	0.312651000	0.000000000
C	-3.565406000	1.487885000	0.000000000
C	-2.827181000	2.698909000	0.000000000
N	-1.489640000	2.411391000	0.000000000
C	-1.273579000	1.032714000	0.000000000
C	0.007625000	0.512318000	0.000000000
C	0.347459000	-0.854117000	0.000000000
C	1.668895000	-1.364949000	0.000000000
C	1.855966000	-2.779272000	0.000000000
C	3.116213000	-3.332980000	0.000000000
C	4.246148000	-2.498136000	0.000000000
C	4.090687000	-1.102878000	0.000000000
C	2.834812000	-0.540550000	0.000000000
H	2.741380000	0.545079000	0.000000000
H	4.986642000	-0.482390000	0.000000000
C	5.629092000	-3.044920000	0.000000000
O	6.624609000	-2.359293000	0.000000000
O	5.656238000	-4.384669000	0.000000000
H	6.592516000	-4.651823000	0.000000000
H	3.248195000	-4.413801000	0.000000000
H	0.977072000	-3.426268000	0.000000000

H	-0.450964000	-1.595622000	0.000000000
H	0.827776000	1.228760000	0.000000000
C	-0.424749000	3.392744000	0.000000000
H	0.203866000	3.274490000	-0.894977000
H	-0.849409000	4.399924000	0.000000000
C	-3.465292000	3.953771000	0.000000000
C	-4.846064000	3.961381000	0.000000000
C	-5.587196000	2.761146000	0.000000000
C	-4.946528000	1.521088000	0.000000000
H	-5.531470000	0.600182000	0.000000000
H	-6.676926000	2.806503000	0.000000000
H	-5.375862000	4.914519000	0.000000000
H	-2.904935000	4.887135000	0.000000000
C	-2.827181000	-0.538441000	-1.271012000
H	-2.137162000	-1.391278000	-1.306145000
H	-3.852259000	-0.935004000	-1.280880000
H	-2.682031000	0.061902000	-2.179774000

Table S1. Calculated HOMO and LUMO values and respective HOMO-LUMO gap for merocyanines **3**, **6**, and **7**.

Merocyanine	Solvent	HOMO (eV)	LUMO (eV)	Gap (eV)
**3**	MeCN	-7.72	-2.53	5.19
	DCM	-7.93	-2.78	5.15
	1,4-Dioxane	-8.80	-3.76	5.04
**6**	MeCN	-8.25	-2.49	5.76
	DCM	-8.49	-2.75	5.74
	1,4-Dioxane	-9.46	-3.79	5.67
**7**	MeCN	-8.22	-2.54	5.68
	DCM	-8.46	-2.80	5.66
	1,4-Dioxane	-9.40	-3.82	5.58

Correlation Data

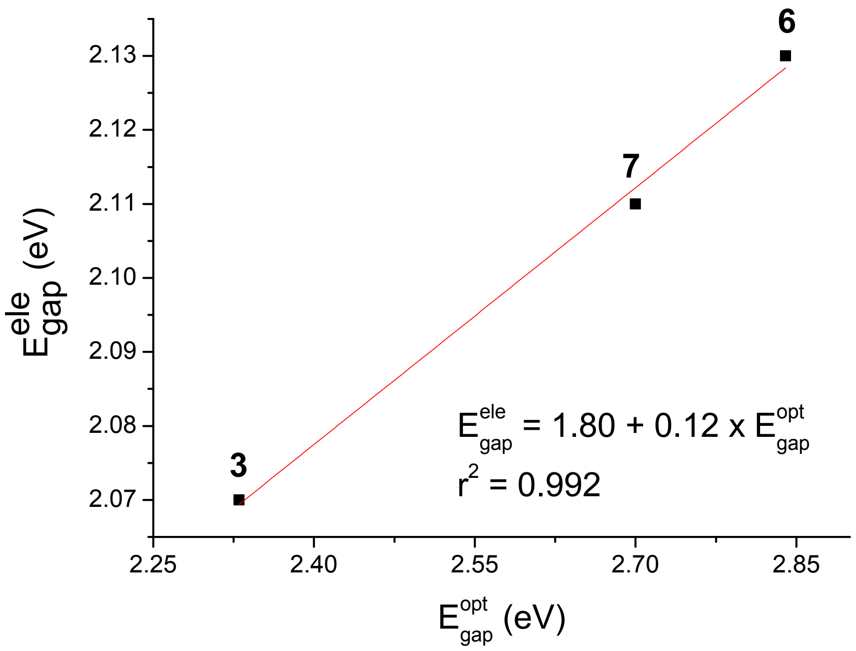

Figure S15. Correlation E^{ele}_{gap} versus E^{opt}_{gap}.

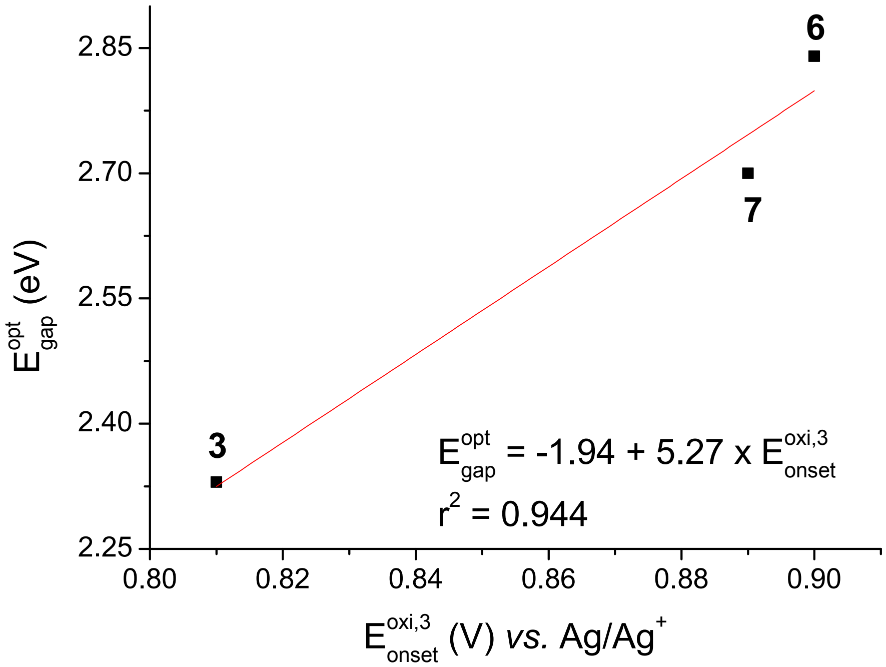

Figure S16. Correlation E^{opt}_{gap} (eV) *versus* $E^{oxi,3}_{onset}$.

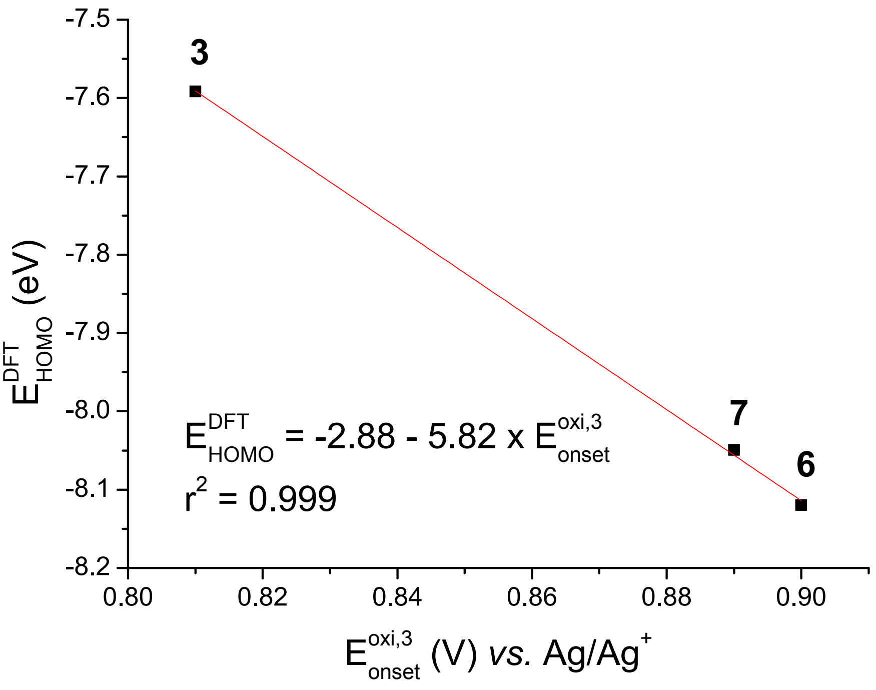

Figure S17. Correlation E_{HOMO}^{DFT} *versus* $E_{onset}^{oxi,3}$.
